# In Which Way Do the Flower Properties of the Specialist Orchid *Goodyera repens* Meet the Requirements of Its Generalist Pollinators?

**DOI:** 10.3390/ijms24108602

**Published:** 2023-05-11

**Authors:** Emilia Brzosko, Andrzej Bajguz, Justyna Burzyńska, Magdalena Chmur

**Affiliations:** Department of Biology and Plant Ecology, Faculty of Biology, University of Bialystok, Ciolkowskiego 1J, 15-245 Bialystok, Poland; j.burzynska@uwb.edu.pl (J.B.); m.chmur@uwb.edu.pl (M.C.)

**Keywords:** bumblebees, floral display, fruiting, nectar amino acids, nectar sugars, pollinaria removal

## Abstract

This article is the next part of a series of studies documenting the influence of flower traits on the reproductive success (RS) of orchids. Knowledge of factors influencing RS helps to understand the mechanisms and processes crucial for shaping plant–pollinator interactions. The aim of the present study was to determine the role of flower structure and nectar composition in shaping the RS of the specialist orchid *Goodyea repens*, which is pollinated by generalist bumblebees. We found a high level of pollinaria removal (PR) and female reproductive success (fruiting, FRS) as well as a high level of variation between populations, although in certain populations pollination efficiency was low. Floral display traits, mainly inflorescence length, influenced FRS in certain populations. Among the flower traits, only the height of flowers was correlated with FRS in one population, suggesting that the flower structure of this orchid is well adapted to pollination by bumblebees. The nectar of *G. repens* is diluted and dominated by hexoses. Sugars were less important in shaping RS than amino acids. At the species level, twenty proteogenic and six non-proteogenic AAs were noted, along with their differentiated amounts and participation in particular populations. We found that distinct AAs or their groups mainly shaped PR, especially when correlations were considered at the species level. Our results suggest that both the individual nectar components and the ratios between them have an impact on *G. repens* RS. Because different nectar components influence the RS parameters in different ways (i.e., negatively or positively), we suggest that different *Bombus* species play the role of main pollinators in distinct populations.

## 1. Introduction

The maintenance of plant species depends on the effectiveness of reproduction in its populations. In animal-pollinated plants, the main determinant of reproductive success is pollinator availability [1,2,3]. Approximately 80% of wild plant species are directly dependent on insect pollination for seed set, and 62–73% of plant populations show pollination limitation [4,5]. One of the group of plants in which pollinator deficiency is recognized as the main factor in low reproductive success is the Orchidaceae family [6]. Specialists appear to be particularly sensitive to pollinator limitations; about 60% of orchids are pollinated by only one pollinator species [7]. Pollinator deficiency has been caused by severe declines in many insect species due to changes in land use and agricultural intensification [8,9,10,11]. Among these are many bees [12], and one of the best documented signs of an ongoing decline in diversity are bumblebees [11], being pollinators of our study object, *Goodyera repens*.

Another cause of pollination limitation in orchids is the low amount of pollen reaching the stigmas [6]. Correct pollen deposition and the appropriate number required to reach effective pollination both depend greatly on mutual matching between flower and pollinator structures [13,14,15,16,17]. The mechanical fit between flowers and their pollinating partners is the result of adaptation of flowers to local pollinators, and is one of the evolutionary mechanisms that shape plant–pollinator interactions [13,14,16]. Pollinators evolve traits to better exploit floral resources in different habitats [5]. The adaptation of pollinators to flowers is especially suggested in cases where the fitness of pollinators strongly depends on their ability to access the reward [18]. The role of flower morphology for the effectiveness of pollination and pollinator-mediated selection is well documented, both in studies in the natural environment and in experiments on flower trait manipulation [15,19,20,21,22,23]. Pollinators are attracted not only by the character of the single flowers, but potentially by the whole inflorescences if plants develop many flowers on one shoot. The importance of floral display (size of flowering shoot, inflorescence, and flower number) for visitation rates and reproductive success have been shown by numerous authors [24,25,26,27,28]. Pollinators are better attracted when the whole community is characterized by a diversity of blooming plants, which represent a wide food offer. The richness of plant species in the neighborhood most often positively influences the pollination effectiveness of a particular species [29,30,31,32,33,34,35], including orchids, especially those which produce rewards and share pollinators [6].

The important flower traits involved in pollinator attraction and pollination efficiency are nectar and its accessibility, quantity, and quality. The role of nectar presence for the reproductive success of orchids has been shown, for example, in the reviews by Neiland and Wilcock [36] and Tremblay et al. [6]. These authors have documented that among the rewarding species, the highest fruiting level is achieved for those that produce nectar. Unfortunately, the nectar chemistry of orchids has very rarely been studied, and even less explored is the problem of the influence of nectar components on their reproductive success. The available data almost exclusively concern the sugar content in nectar [37]. A meta-analysis by Brzosko and Mirski [37] has shown that most orchids produce sucrose-dominant nectar, which is consistent with the common assumption that floral nectar in Angiosperms is most often sucrose-dominant [38,39,40]. Brzosko and Mirski [37] documented a clear relationship between sugar concentration in nectar and types of orchid pollinators. The highest concentration of nectar in orchids is characterized by bee-pollinated species. Pollinator preferences for nectar concentration and composition have been demonstrated in studies of many plants from different taxonomic groups [38,40,41,42,43,44]. These preferences are mainly related to dietary requirements and types of mouth apparatus adapted to remove nectars of different accessibility, viscosity, and composition. For example, it is known that large pollinators need more energy than smaller ones due to the costs associated with their body size and behavior; therefore, they feed on plants that produce larger amounts of more concentrated nectar [45,46]. On the other hand, insects that have long proboscises feed more often on diluted nectar due to its lower viscosity being easier for sucking; moreover, only such insects can use nectar accumulated in spurs and long tubes [47].

The other important components of nectar shaping plant–pollinator interactions are amino acids [38,42,48]. Their role has been less studied than that of sugars; nevertheless, it is known that, as a source of nitrogen for pollinators, they are crucial for their growth, development, and reproduction. Insects that feed on nectar rich in amino acids are larger, have greater longevity, and produce more offspring with greater fitness [49,50]. Amino acids can determine pollinator behavior, attracting or deterring flower visitors and thereby influencing plant reproductive success. The differentiated gustatory and stimulatory needs of pollinators cause plants to produce nectar directed to a given type of pollinating partners [49,51]. Previous studies have documented pollinator preferences for the total amount of AAs in nectar as well as for particular AAs [47,52,53,54]. For example, butterflies prefer nectar rich in AAs, whereas birds and flies choose nectars with lower concentrations [51,55].

Studies have reported variation in flower traits in the geographic range of species, which is the consequence of adaptation to local pollinators, mainly the most effective [20,56,57,58,59,60,61,62]. Pollinator-mediated selection of flower traits is one of the main evolutionary mechanisms shaping plant–pollinator interactions [13,16,63,64]. The spatial variation of the relationships between both groups of organisms translates into differentiation of the reproductive success of plants [5,8,19,61,65].

The aim of the present study was to explain the way in which small flowers of *G. repens* (specialist) are adapted to its large pollinators, namely, bumblebees (generalists). This is an interesting model to use in investigating the relationship between specialist plant species and generalist pollinators. We tested the importance of flower structure and nectar chemistry for the level of reproductive success (pollinia removal and fruit set) in eight populations of *G. repens* from NE Poland. Our studies fit into existing problems concerning evolutionary processes and the mechanisms shaping plant–pollinator interactions.

## 2. Results

### 2.1. Floral Display and Flower Structure

Among floral display traits, only shoot height (SH) varied significantly between *G. repens* populations (F = 6.69; *p* = 0.0). The value of this trait was the lowest in the FORT and the highest in the WIG population (Table 1). The length of the inflorescence (IL) and number of flowers (NF) were similar in all populations (F = 1.91; *p* = 0.07 and F = 0.69; *p* = 0.67, respectively; see Table 1). The values of all measured flower traits differed significantly between *G. repens* populations (F = 4.33–11.24; *p* < 0.001), and the smallest flowers were observed in two populations located in the Białowieża National Park, BIA1 and BIA2 (Table 1).

### 2.2. Nectar Chemistry

#### 2.2.1. Sugars

**Figure 1:**
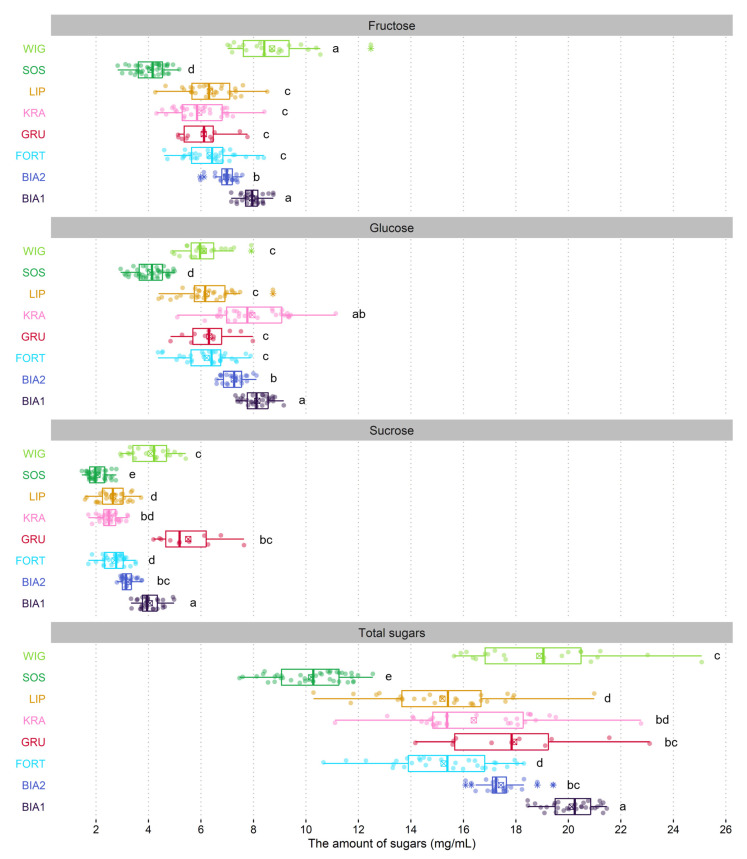
Boxplots of sugar amounts for populations of *Goodyera repens*. The colored dots are individual samples. The square crosses shows the mean. The lower and upper hinges correspond to the lower (Q_1_) and upper (Q_3_) quartiles. Thus, the length of the box shows the interquartile range (IQR). The thicker line inside the boxes corresponds to the median. The lower whisker extends from the hinge to the smallest value (at most Q_1_ − 1.5 × IQR) of the hinge. The upper whisker extends from the hinge to the highest value (no further than Q_3_ + 1.5 × IQR). Data beyond the end of the whiskers, indicated with an asterisk symbol, are outliers. Different lowercase letters indicate statistically significant differences according to the pairwise Wilcoxon rank sum test with Benjamini–Hochberg adjustment (*p* < 0.05).

**Figure 2:**
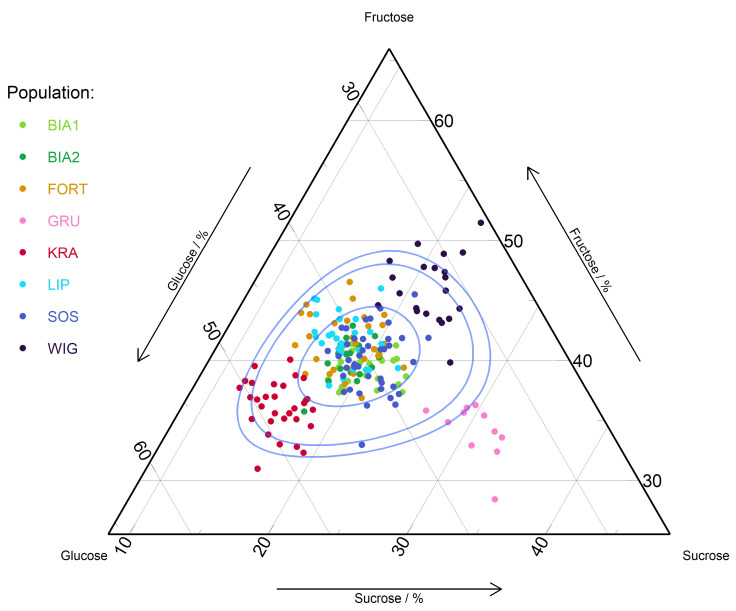
Ternary plot of sugars for populations of *Goodyera repens*. The blue lines show the 50%, 90%, and 95% confidence intervals using the Mahalanobis distance and the log-ratio transformation.

The sum of sugars in the nectar of *G. repens* differed significantly between populations (F = 396.39; *p* = 0.0); the lowest was in SOS (10.2 ± 0.2 mg/mL) and the highest (almost two times higher than in SOS) was in WIG and BIA1 (Figure 1 and Figure 2). The nectar of *G. repens* is dominated by hexoses in all populations (69.4 ± 2.5%–84.6 ± 2.0%), and the ratios of hexoses to sucrose ranged from 2.2 in SOS and GRU to 5.1 in KRA. We found differentiation in the glucose-to-fructose ratio between populations (F = 85.24; *p* = 0.0). In KRA glucose were more abundant, while in WIG fructose was (glucose-to-fructose ratios were 1.4 and 0.7, respectively), while in the remaining populations the amount of these sugars was balanced (Table 2).

#### 2.2.2. Amino Acids

The total amount of AAs in *G. repens* nectar differed significantly between populations (F = 510.405; *p* = 0.0) and ranged from 242.8 μM in the LIP population to 989.3 μM in the BIA2 population (Table 3). At the species level, twenty proteogenic and six non-proteogenic AAs were noted. All of them were found in two populations (SOS and KRA). Cit and BABA were absent in the WIG and LIP populations, while BABA was absent in SOS and AABA was absent in GRU. Orn was not detected in the nectar of either population in Białowieża National Park (BIA1 and BIA2). It is interesting that β-Ala and Nva were absent in *G. repens* nectar. We found statistically significant differences in the participation of all AAs between populations (F = 182.03–2404.18; *p* = 0.0). Their lowest percentage ranged from only a few to about ten times lower than the highest. The most abundant AA was Asp; its participation in the SOS, GRU, and LIP populations equaled about 22%, while in the remaining populations it ranged from 6.9% to 15.2%. Other relatively abundant AAs were Gly (4.1–16%) and Gln (3.3–15.4%).

**Figure 3:**
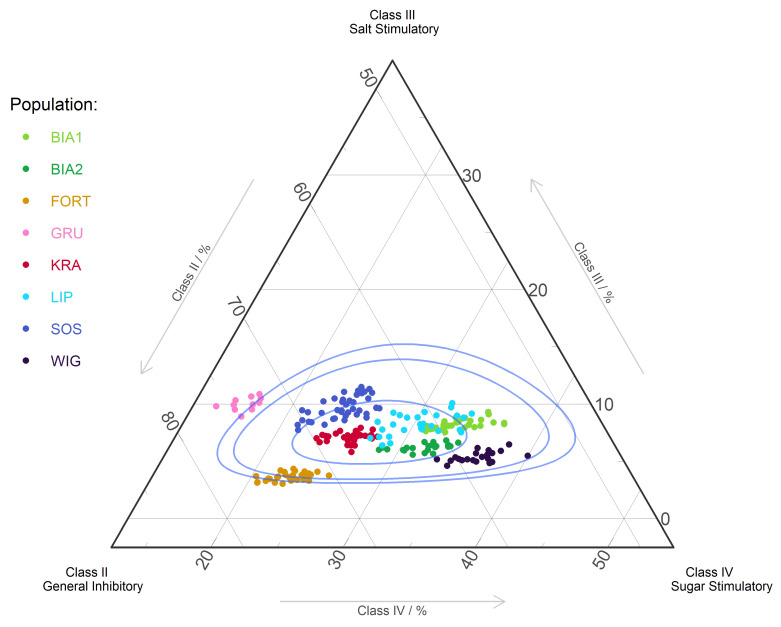
Ternary plot of amino acid taste classes II–IV for populations of *Goodyera repens*, representing the impact of AAs on insect chemoreceptors. The blue lines show the 50%, 90%, and 95% confidence intervals using the Mahalanobis distance and the log-ratio transformation. The first class of AAs (Asn, Gln, Ala, Cys, Gly, Ser, Thr, and Tyr) does not affect insect chemoreceptors.

We observed statistically significant differences in the participation of proteogenic and non-proteogenic AAs (F = 408.79; *p* = 0.0; 254,593; *p* = 0.0, respectively). Furthermore, we found variation in proteogenic to non-proteogenic AAs ratios, from 10.9 in LIP to about 24 in the BIA2 and GRU populations. The populations differed in the participation of EAAs and nEAAs as well. In seven populations we observed the dominance of nEAAs (2.2–4.4 times), while in WIG the EAAs were more abundant (nEAAs:EAAs = 0.9). In addition, we found differentiation in the participation of AAs belonging to different taste groups (F = 329.370–588.537; *p* = 0.0), with the most abundant being AAs from class II (Figure 3).

### 2.3. Reproductive Success

We observed differentiated level of RS in the populations of *G. repens*. The lowest PR was found in the SOS (51.6 ± 4.1%), and in the remaining populations it ranged from 67.7 ± 5.3% to 78.7 ± 4.1% (Table 4). On the other hand, FRS was the lowest in FORT (56.1 ± 5.3%), while in the other populations it was from 65.1 ± 9.2% to 78.1 ± 4%. We observed variation in pollination efficiency among populations. In three populations (GRU, KRA, and SOS), the PR to FRS ratio equaled 1–1.1, while in three other (FORT, WIG, and LIP) it ranged from 1.5 to 4.

### 2.4. Determinants of Reproductive Success

Floral display traits influenced only FRS (Table 5, Figure S1). In four populations (FORT, SOS, BIAL1, and LIP) the fruit set was positively but weakly correlated with the inflorescence length (r_S_ = 0.37–0.49, *p* < 0.05), and in BIA1 it was correlated with shoot height (r_S_ = 0.60, *p* < 0.05). The number of flowers had a negative impact on fruiting in SOS (r_S_ = −0.35, *p* < 0.05). In the WIG, GRU, and KRA populations, floral display traits did not influence reproductive parameters. Among flower traits, only FH in SOS influenced FRS (r_S_ = 0.47, *p* < 0.05). At the species level, height of shoots and inflorescence length had an impact on FRS (r_S_ = 0.25 and r_S_ = 0.24, *p* < 0.05, respectively).

Sugars had a week impact on RS in *G. repens* populations; the percentage of glucose positively correlated with FRS in FORT and fructose was negatively correlated with FRS in WIG (see Table 5, Figure S1). Sugar concentration negatively influenced PR and FRS in WIG (Table 5). We found a stronger impact of nectar components on RS in the case of AAs, especially in KRA, where twelve AAs were positively correlated with PR (Table 5, Figure S1). Interestingly, fruiting in this population did not depend on any of the AAs. In the remaining populations, one to a few distinct AAs influenced RS, both positively and negatively (Table 5, Figure S1). In two populations, we found statistically significant correlations between the total amount of AAs and RS—in BIA1 with FRS (r_S_ = 0.46, *p* < 0.05), and in KRA with PR (r_S_ = 0.53, *p* < 0.05). The sum of non-protein AAs positively influenced PR in FORT and KRA and negatively influenced FRS in FORT. In BIA1, fruiting was positively influenced by the participation of non-essential AAs, negatively influenced by the participation of essential AAs (r_S_ = 0.66 and r_S_ = −0.59; *p* < 0.05; respectively), and positively influenced by the relationship between them (r_S_ = 0.67, *p* < 0.05) (Table 5). In KRA, both essential and non-essential AAs positively influenced PR. In six populations, RS was influenced by AAs from different taste groups (Table 5, Figure S1). In FORT, we found a dependence of PR on the ratio between sugars and non-essential AAs (r_S_ = −0.42, *p* < 0.05).

When we considered correlations between the parameters of RS and nectar components at the species level (pooling all data), we found that the sum of sugars, their concentration, and the amount of particular sugars positively influenced PR, while all of these parameters were correlated with FRS (Table 5, Figure S1). In addition, we noted the influence of the amount and/or percentage of 25 AAs on PR, while only four of them influenced FRS (Pro positively and Leu, Orn, and GABA negatively). Among statistically significant correlations between floral traits and reproductive parameters, 64 concern PR and 8 concern FRS.

## 3. Discussion

Plants have evolved a wide range of strategies to achieve the highest possible level of reproductive success. One of their powerful strategies is the production of rewards, among which nectar is the most effective. For example, nectar-rewarding orchids are more successful in setting fruits than nectarless ones [6,36]. As in the case of other nectariferous orchids, we observed a high level of RS in *G. repens* populations. Variation of fruiting in different parts of the species range was found in [36,66,67]. Spatiotemporal variation in FRS (from 25% to 83.6% in different populations and years) was noted by Brzosko et al. [68]. The storage of nectar in open nectary increases its availability for visitors, and as such may influence the RS of this orchid. Bumblebees (*Bombus terrestris*, *B. pascuorum*, and *B. lapidarius*) are recognized as *G. repens* pollinators, although in a large population in Germany the main pollinators were honeybees [66]. Bumblebees are known to be efficient pollinators, as they can visit many flowers in a short time and are able to fly significantly further distances than other insects [66,69,70,71]. Distinct *Bombus* species differ in their foraging range. Knight et al. [69] found that *B. terrestris* has the greatest foraging range (758 m), while *B. pratorum* operates at 674 m and *B. lapidarius* and *B. pascuorum* at about 450 m. Chapman et al. [72] estimated that they fly even further, *B. pascorum* up to 2.3 km, and *B. terrestris* up to 2.8 km. In effect, the traveling ability of particular *Bombus* species may influence visitation rate, and thereby the level of *G. repens* RS. Bumblebees fly at relatively long (but still restricted) distances. Therefore, large forest areas may be an important barrier for them when moving from one source of food to another, especially for species that fly at shorter distances from their nests [70]. In addition, pollinator abundance may be restricted in shaded habitats, in which *G. repens* grows [66], as most *Bombus* species nests are located in open areas. The level of RS of *G. repens* is shaped by pollinator abundance as well as by their behavior. Bumblebees have the ability to learn, which is important for the pollination efficiency of *G. repens*, as only a few flowers in the inflorescence open simultaneously. In such a case, repeated visits are needed in order for the orchid to achieve the maximum possible pollination success, although Claessens and Kleynen [66] reported that, unlike other pollinators, bumblebees rarely visit the same plant. Although a generally high level of RS was observed, in certain localities PR and/or FRS was lower than in others, which might suggest differentiated pollinator abundance in particular populations. This could have a number of causes. One may be the scarcity of bumblebee colonies in the vicinity of orchid populations, as they are localized in large forest complexes. *Goodyera repens* is often only one of many flowering plants in the undergrowth of coniferous forests, and its populations may be too small to be fully detected by pollinators, especially if their nests are located far away. Low resources (nectar) in such populations may be inadequate to the pollinators’ energetic costs connected with far flight distances; therefore, after a first visit they may decide to restrict renewed visits. The frequency of visitation rate in *G. repens* may depend on the presence and abundance of other species which attract these pollinators. Usually, one co-flowering plant during the blooming time of *G. repens* in coniferous forests is *Melampyrum pratense*, which also depends on bumblebees, predominantly on short-tongued species [73]. The presence of *M. pratense*, as well as other co-flowering plants, can act in two ways on orchid RS, facilitating visitation rate and as a result increasing RS, or competing for pollinators, which can lower RS [29,32,33,34], especially when pollinators are not abundant. The lack of *M. pratense* in the vicinity of the LIP population could be one of the causes of lower fruiting and PR in this location. In the same way, it could be explained the lower PR in SOS and FRS in FORT, where single shoots of *M. pratense* were noted. This suggests that plants co-flowering with *G. repens* have a positive impact on its RS. The positive influence of *M. pratense* on RS was suggested by Brzosko et al. [61] in the case of *Epipactis helleborine*, for which bumblebees are important pollinators and which exists in the same forest as the SOS *G. repens* population. However, Claessens and Kleynen [66] reported that bumblebees are regular visitors to *G. repens* despite the lack of other plants due to the large supplies of nectar in orchid flowers.

An important part of the plant strategies on which RS depends is the flower structure, which is adapted to the size and behavior of pollinators. The mechanical fit between flower and pollinator ensures correct flower penetration, and is the basis of effective pollination [14]. The impact of flower traits on RS has been observed in many studies [13,15,16,21]. Generally, the mechanical match between plants and pollinators is stronger in specialized systems [74]. An example of such a relationship could be *G. repens* and bumblebees. *Goodyera repens* flowers do not open very wide (the sepals and petals form a narrow tube), and newly opened flowers open only slightly, leaving little space between the lip and viscidium [66]. While this restricts the penetration of the flower by many insects, the rigid proboscis of the bumblebee is well suited to attachment of the viscidium to the adhering pollinia [66]. The match between *G. repens* flowers and its pollinator may reflect a relatively high level of RS; in most populations, both PR and FRS were higher than these observed for nectariferous orchids [6,36]. The near lack of correlations between flower traits and RS may indicate that the flower structure is well adapted to pollination by bumblebees. A weak influence of flower traits on RS has been found in other orchids as well [37,61,62]. Although levels of both PR and fruiting were high, pollination efficiency in three populations (FORT, WIG, and LIP) was low (PR:FRS ranged from 1.5 to 4). First, this may indicate a deficiency of pollinators. Second, low fruit-to-flower ratios in many plants may be an effect of the paucity of resources available for fruit development, as per the resource-limitation hypothesis [6]. The RS of the plants may depend on the characteristics of the floral display. As the higher plants are more visible, they are better able to attract pollinators which visit more flowers, thereby increasing fruiting [25,26,27,28,61,75]. Floral display traits influenced only FRS in *G. repens* populations. In four of them, the fruiting was higher on longer inflorescences. On the other hand, higher shoots seemed to be preferred only in BIA1, which could indicate that the flowering shoots of this orchid, as the only or almost only flowering species in the ground layer at this time, are well recognized by pollinators independently of their size. This agrees with the finding that stronger pollinator-mediated selection with respect to shoot size is noted more in taller than in shorter vegetation [76,77,78]. The negative correlation between flower number and FRS in SOS confirms, as suggested above, that there is a pollinator deficiency in this location. In the case of *G. repens*, the fact that only few flowers open simultaneously is non-negligible for the RS, which may influence its attractiveness for pollinators [8]. The lack of differences between populations in terms of inflorescence length and flower number, along with scarce correlations between floral display and RS parameters, may indicate similar reproductive potential of each population, and suggests that other factors are more important in shaping the RS of this orchid.

One of the key problems in understanding plant–pollinator interactions and coevolution is knowing the preferences of pollinators with regard to nectar composition. Nectar is an important energy source for many animals [38,40,43], and can manipulate pollinator behavior [42,79,80,81]. In effect, nectar can influence the RS of plants. Different pollinators show preferences for different nectar traits (i.e., volume, concentration, and particular components), and nectar properties are connected with pollinator types, representing different mouth apparatuses, dietary needs, and behavior [41,43,47,82]. The best studied nectar components in the context of pollinator requirements are sugars. Among known pollinators, bees prefer the highest sugar concentration in nectar [41,43,83,84]. Moreover, bee-pollinated orchids are characterized by more concentrated nectar (c.a. 40%) than species pollinated by other animals [37]. Pouvreau [85] found that sugar uptake by foragers of *B. hypnorum*, *B. lapidarius*, and *B. pratorum* was the highest for sugars at 30–40%. The experimental study of Cnaani et al. [86] showed that *B. impatiens* prefers more concentrated than diluted sugar solutions (40% vs. 13%). Brown and Brown [87] found that *B. terrestris* prefers even more concentrated nectar (50–60%). Pamminger et al. [84], analyzing the results of experimental and field studies, concluded that a sugar concentration below 20% is of low quality for bees, while 20–35% is adequate and 35–65% is optimal. In light of these results, it is surprising that *G. repens*, a specialist adapted to pollination by insects which require a high sugar concentration due to their high energetic costs, produces diluted nectar (5.3–10.2%). Our results fit better with studies reporting lower nectar concentrations in bee-pollinated plants [88,89]. Sugar concentration is considered a key trait that influences attractiveness, as it determines the energetic value of nectar. However, a higher concentration of nectar increases its viscosity, making nectar difficult to use by some insects and more energy- and time-consuming. To this point, pollinators choose nectar that optimizes the balance between optimal concentration (energetic needs) and optimal viscosity (energetic costs) [90].

This is an interesting way to explain why *G. repens*, which depends only on bumblebees, offers them nectar with a concentration below their energetic needs. The most simple explanation could be the suggestion of Nicolson and Thornburg [38] that nectar sugar composition is determined more by phylogeny than by pollinator preferences. This explanation may explain the low nectar concentration in other orchids [59,60,61,62]. Another possible explanation could be that bumblebees mainly rely on other plants as their main food, with this orchid being a minor component of their diet, especially when its populations are small. The presence of *M. pratense* in the vicinity of the orchid may compensate for the low nectar concentration in *G. repens*. *Melampyrum* nectar is more concentrated, with sucrose participation of ~40% [91], and is a valuable pollen source. However, in penetrating forests, in which *G. repens* grows, bumblebees have a restricted offer; the orchid is often the only flowering plant. Thus, nectar of a lower quality is better than nothing. *Goodyera repens*, as the only flowering species, does not need to invest in the production of highly concentrated nectar, which might be a part of its life strategy. Another cause of the low sugar concentration of *G. repens* nectar might be related to the character of its habitats. High-quality nectar production is costly, and this orchid grows in relatively poor habitats. Its short rhizomes are shallowly rooted in mosses and the upper organic layer; thus, *G. repens* cannot use nutrients immediately from the soil. Cameron et al. [92] found that C and N assimilated by a fungus are transferred to the roots and shoots. Thus, the activity of the mycorrhizal partner plays an important role in the supplementation of carbon necessary for the production of sugars.

Apart from nectar concentration, sugar composition may be adapted to the main pollinators, and the literature presents a number of general trends associating nectar sugar composition with pollinator groups. Data concerning the preferences of bumblebees for the three main sugars are not consistent; additionally, distinct species may favor different sugars. For example, Mommaerts et al. [93] and Hendriksma et al. [94] found a strong preference for sucrose-rich nectar. Peter and Johnson [95] suggested that their preferences may vary with regard to low and medium sugar content, as they are a heterogeneous group. *Goodyera repens* nectar is dominated by hexoses in all populations (69.4–84.6%), and the ratios between hexoses and sucrose are different (2.2–5.1). Our results are consistent with the finding of Baker and Baker [82] that short-tongued bees (the main *G. repens* pollinators) prefer hexose-rich nectar. In addition, Petanidou [44] noted a preference of short-tongued bees for fructose in nectar of plants in phrygana communities. Other authors have found that distinct *Bombus* species favor different sugars. For example, Pouvreau [85] found that short-tongued *B. terrestris* favors sucrose, while long-tongue *B. hypnorum* favors glucose. The same author points out that bumblebees prefer nectar that is an equal mixture of sucrose, glucose, and fructose over nectar dominated by one sugar type.

From the point of view of plant–pollinator interactions, the important components of floral nectar are the AAs. They are sources of diet, and can influence pollinator behavior according to the ‘manipulation hypothesis’ [42,46,96]. As with sugars, their amount and composition may be related to particular groups of pollinators [51,55,81,97]. Several of these, such as butterflies, select nectar with a high amount of AAs, while birds and flies prefer lower concentrations [51,55]. Petanidou [43] found that in phrygana communities AAs play a more important role as phagostimulants than sugars. Bumblebee preferences with respect to AA content are not consistent, although they can differentiate certain AAs and their concentration, as they have receptors which help them to assess the AA content prior to consuming food [98,99]. Stabler et al. [99] found that high AA intake is associated with poor survival of bumblebees, while Ruedenauer et al. [100] did not observe such a negative effect. On the other hand, Archer et al. [12] showed that a high intake of AAs increased body mass of *B. terrestris*. In our studies, the total amount of AAs positively influenced FRS in BIA1 and PR in KRA, where the content of AAs in nectar was one of the highest. It is interesting that, in KRA (the population with the second highest total amount of AAs), PR was correlated with sum of AAs. We observed the influence of twelve distinct AAs on PR in KRA, while in the BIA1 (with the highest amount of the AAs) all of the AAs were correlated with PR. This suggests that distinct bumblebees with distinct preferences for particular AAs are the main pollinators in these two populations. When we tested the relationships between the total amount of AAs and RS at the species level (data were pooled), we found that the increase in total AAs caused an increase of PR. This may indicate that *G. repens* pollinators need a large amount of AAs, which contrasts with the results of Stabler et al. [99]. The most abundant AA in *G. repens* nectar was Asp, one of the most abundant AAs in the nectar of other orchids [59,60,61,62]. Asp influences pollinator behavior, and is known as a general repellent [101]. In *G. repens*, the amount of Asp was positively correlated with FRS in BIA2, with the lowest participation of this AA, and with PR in KRA. At the species level, the higher amount of Asp caused an increase in PR, while its higher percentage caused a decrease in PR. The two other AAs with relatively high participation were Gln and Gly. Gln is one of the most common AAs in *E. palustris* nectar [60]. Gln is needed for energetically exhaustive flights [101], and Gly provokes a feeding response in honeybees [102]. The Gln amount positively influenced PR only in KRA. At the species level, Gln was positively correlated with PR and Gly was negatively correlated with PR. Hendriksma et al. [103] found that nectar enriched with Gly significantly deterred bees. In certain populations of *G. repens*, we observed relatively high participation (~10%) in the nectar of AAs such as Glu, Ala, Ser, Asn, and His. Glu, one of the most abundant AAs in the nectar of other orchids [60,61,62], affects pollinator behavior, helps in parasitoid rejection, and is needed for energetically exhaustive flight [38,51,104,105,106]. A higher amount of Glu increased PR in KRA. Ser influences growth and behavior [51,105], although Bertazzini et al. [107] reported a negative response of honeybees to this AA. The amount of Ser positively influenced PR in FORT and negatively influenced PR in GRU. At the species level, Ser positively influenced PR. Ala influences insect growth, and may deter honeybees [35]; however, in our studies it did not influence RS in any population, similar to His, which also had large participation in certain populations. Both Ala and His positively influenced PR at the species level. It is worth mentioning the negative influence of Tau on FRS in the FORT, BIA1, and BIA2 populations and its positive impact on PR in KRA, although at the species level this AA negatively shaped PR. It is worth highlighting the highly differentiated role of distinct AAs in shaping RS in particular populations. One of the best examples demonstrating this variation is the impact of twelve AAs on PR in KRA, while in other populations only one or a few AAs were correlated with PR. On the other hand, in two populations in Białowieża National Park (BIA1 and BIA2), eight AAs influenced fruiting, while in the remaining populations FRS did not depend on AAs or was influenced only by single AAs.

Previous studies of nectar components have pointed out the importance of nonprotein AAs, although the role many of them is unknown. Different functions are attributed to this group, mainly connected with the discouragement of inefficient pollinators and influence on pollinator behavior [108,109,110]. For example, *B. terrestris* showed changes in walking and flying after consumption of non-proteogenic AAs, although the effects varied with composition and concentration [109]. In *G. repens* nectar, six nonprotein AAs were found with differentiated participation in particular populations. Their total amount positively influenced PR in FORT and KRA and negatively influenced FRS in FORT. For several (Cit, Orn, Tau, BABA, and GABA) we observed correlations with RS parameters in four populations, which were always positive with PR and always negative with FRS. Gardener and Gillman [51] suggested that Tau, Orn, and GABA affect the neurobiology of insect appetite. GABA accumulates in response to infection by bacteria and fungi, and bumblebees are among those insects associated with a higher concentration of GABA in nectar [110]. In addition, GABA acts in synergy with Tau, which in our studies negatively influenced FRS in three populations and positively influenced PR in one population. At the species level, a higher participation of Tau decreased PR and did not have an impact on FRS. BABA plays a similar role as GABA, and was absent in three populations. Bogo et al. [109] found an increase in longevity of *B. terrestris* that fed on nectar with a high concentration of GABA. Among the most common AAs in floral nectar is Pro, which triggers salt receptors in insects and leads them to initiate feeding [47,111], rewards pollinators, and acts as a propellant for the lift phase of flight [110,112]. Carter et al. [112] suggested that accumulation of Pro is an answer to stress factors in plants. The preference of honey bees for proline-enriched nectar was noted by Bertazzini et al. [107], while Teulier et al. [113] found that bumblebees use Pro in the early phase of flight. Pro was observed in all *G. repens* populations, with quite a high amount in several of them, but only positively influenced PR in KRA, and FRS in WIG. It is interesting that at the species level the percentage of Pro was negatively correlated with PR and positively correlated with FRS. The absence of β-Ala in *G. repens* is surprising, as it is one of the most abundant non-proteogenic AAs in floral nectar and plays an important role in plant metabolism while attracting pollinators and stimulating flight muscle function [97,110]. The lack of this AA is strange, especially in light of the importance of this AA documented in the review of Barberis et al. [108] and of the finding of Bogo et al. [109] that *B. terrestris* prefers a solution enriched with β-Ala.

The correlations, or lack thereof, between particular nectar components and plant RS can only partially explain the relationships between plants and pollinators. Archer et al. [12] suggested that the way in which AAs interact with each other to influence fitness is not known. This suggestion confirms the report of Nepi [110] that maintaining a high insect feeding rate is an effect of the combined phagostimulatory activity of Pro, Phe, and GABA. Experimental studies have documented that the reaction of bumblebees to certain AAs is connected with the concentration of other nectar components, for example, sugars [109].

An interesting founding of our studies is that positive correlations were observed between the parameters of RS and the amount of particular nectar components or their groups in many cases, as well as in the ratios between them, while negative correlations were simultaneously observed between their percentage and RS. This suggests that the ratios between particular nectar components may be more important in shaping plant–pollinator interactions than their absolute amount. The importance of balance in pollinator diets to ensure their fitness and survival is supported by other authors in the case of bumblebees [12,96,99,110,114,115]. In many insects, consumption of moderate to high ratios of proteins to carbohydrates increases female fecundity [12,114,116]. Stabler et al. [99] found that interaction between the AA mix and the dietary AA:C ratio affects the survival of *B. terrestris*, and that a diet high in EAAs caused a higher rate of mortality in adult workers of these insects. They found that *B. terrestris* fed on a diet with an EAAs:C ratio less than 1:90 (*mol*/*mol*) had a three to seven times greater risk of dying than those fed on a diet higher in carbohydrates, e.g., >1:90. Paoli et al. [114] noted reduced survival in honey bee workers fed on a diet high in EAAs. It is important that nutrient regulation strategies may depend on the source of dietary nitrogen [99]; when casein was a protein source, *B. terrestris* chose food with a 1:149 (*w*/*w*) protein to carbohydrate (P:C) ratio, while when feeding on an equimolar AA mix they chose an 1:560 (*w*/*w*) EAAs:C ratio. On the one hand, these ratios in *G. repens* (C:AAs: 145:1–450:1 and C:EAAs: 598:1–1585:1) appear to be suitable for the survival of bumblebees in light of the results of Stabler et al. [99]. On the other hand, negative correlations between the C:AA and C:EAA ratios and PR at the species level may suggest a deficit of AAs in *G. repens* nectar. Moreover, these ratios were in many cases higher than in other studies, and were greatly differentiated between populations; thus, the question arises as to whether all of them are adequate for the survival and other aspects of the life strategy of *Bombus* species. The answer to this question requires detailed experimental studies in which the diet should be composed in the same way as it is present under natural conditions. The importance of the more comprehensive impact of combined AA compositions on RS suggests correlations between taste groups and reproductive parameters. It is known that nectar taste can attract or deter flower visitors [38,51,104,110]. The preference for a given taste can be based on concentration [40]. An overabundance of certain AAs changes the taste of nectar, which may not be preferred by insects; thus, they may avoid flowers with such nectar. We found an influence (both positive and negative) of different taste groups on PR and FRS in six populations. Furthermore, the impact of AAs of all taste groups was noted at the species level. This suggests that bumblebees are sensitive to the taste of nectar produced by *G. repens*, which reflects the levels of RS in particular populations, although they may react differently. Assuming the ability of bees to taste amino acids [99,103], we suggest that different *Bombus* species with different preferences with regard to nectar taste are responsible for pollinating *G. repens* flowers in particular populations.

## 4. Materials and Methods

### 4.1. Study Species

*Goodyera repens* (L.) R.Br. (Orchidaceae) covers a large disjunctive area, and its geographical range includes central, northern, and eastern Europe, the Caucasus, and partially east Asia, as well as boreal and mountainous areas of North America [117,118]. In Poland, *G. repens* occurs mainly in the north and northeast of the country and in the mountains. This is related to the area of coniferous species, which prefer moderately moist habitats. It is a clonal orchid with evergreen monopodial shoots. The plants are about 10–20 cm high and have rosettes with oval leaves. The stem is hairy, and the small creamy-white flowers are arranged in a single spiral row and emit a sweet scent [66,118]. The perianth is covered with long whitish hairs. The sepals and petals form a narrow tube; thus, the flowers do not open very wide. The lip has two parts, a pointed and triangular gutter-shaped epichile and a round sack-shaped hypochile where nectar is secreted. The column consists of a pollinarium with two yellow pollinia and a large round stigma. The main pollinators of *G. repens* are bumblebees, mainly *Bombus terrestris*, *B. pascuorum*, and *B. lapidarius* [66]. Eichmann and Kozina [119] found that other effective pollinators include *B. pratorum*, *B. hypnorum*, *B. hortorum*, *B. subterraneus*, and *B. lucorum*. The rigid proboscis of bumblebees is well suited to attachment of the viscidium to the adhering pollinia. Bumblebees start their visits from the bottom of the inflorescence and creep upward, inspecting all open flowers. They can visit many flowers in a short time, though they rarely revisit a particular plant [66].

### 4.2. Fieldwork and Floral Trait Measurements

**Figure 4:**
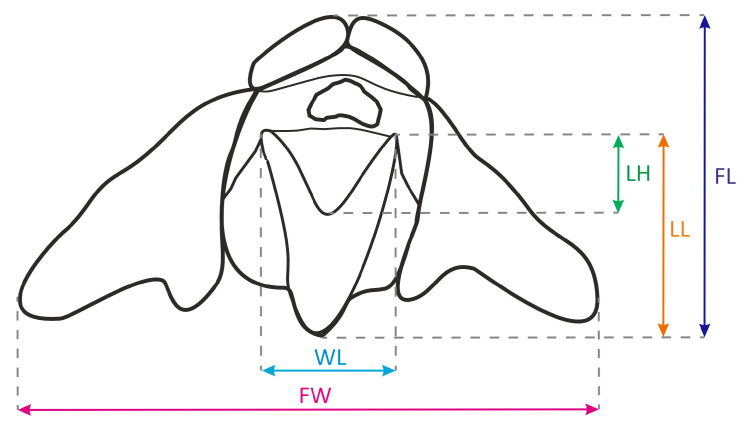
*Goodyera repens* flower diagram, showing measured parameters. Abbreviations are presented above in the text.

Our investigations were carried out in July and August of 2022 and examined eight *G. repens* populations in NE Poland. All of them were located in coniferous forests with *Pinus sylvestris* domination and with poor floral composition in the undergrowth. In different populations, 13 to 42 flowering individuals were marked, depending on population size. In total, 197 plants were included in the study. The flower display traits (shoot height, inflorescence length, and number of flowers) were quantified in the field. In sunny and hot weather, the few fresh unpollinated flowers from the lower part of each inflorescence were collected depending on the size of the inflorescence and the number of open flowers. All of them were used for the analysis of nectar composition, with values of nectar parameters provided per flower. The two lowest flowers were used for the measurement of morphological variables (i.e., flower lenght, FL; flower width, FW; length of labellum, LL; width of labellum, WL; and length of hypochile, LH) (Figure 4) using a DSX110 optodigital microscope (Olympus Life Science, Waltham, MA, USA). The flower traits of an individual plant are provided as the average of two measurements.

To assess the level of reproductive success (RS), the number of flowers on the marked shoots was counted. During capsule maturation, male reproductive success (pollinaria removal, PR) and female reproductive success (fruiting, FRS) were quantified. FRS was evaluated as the number of fruits to the number of flowers in the inflorescence. PR was determined as the number of pollinaria removed to the total number of pollinaria for each inflorescence. Both FRS and PR are provided as percentages. The efficiency of pollination in particular populations was calculated as the ratio of PR to FRS, with a higher index value indicating lower pollination efficiency. To asses RS, only these flowers which remained on the shoots after collection of flowers were included in our morphological and chemical analyses.

### 4.3. Nectar Analysis

#### 4.3.1. Nectar Isolation

Flower nectar isolation was performed using a water washing method. Flowers per sample were placed in a 2 mL Eppendorf tube containing 1 mL of distilled water and shaken in a laboratory thermomixer (Eppendorf Corporate, Hamburg, Germany) at 120 rpm and 21 °C for 45 min for nectar efflux. The flowers were removed from the tubes and the mixture of water and nectar was evaporated dry at 45 °C using a centrifugal vacuum concentrator (Labconco CentriVap Micro IR, Kansas City, MO, USA). The obtained pellet was dissolved in 20 µL of distilled water, then transferred to an MPW-55 centrifuge tube with a filter (MPW Med. Instruments, Gliwice, Poland) and centrifuged to remove impurities at 9000× *g* for 5 min. The purged extract was collected in a glass vial with a 250 µL insert and polymer feet.

#### 4.3.2. Sugar and Amino Acid Determination

Determination and quantification of sugars and AAs were performed using the high-performance liquid chromatography (HPLC) method. An Agilent 1260 Infinity Series HPLC apparatus (Agilent Technologies, Inc., Santa Clara, CA, USA) with quaternary pump with in-line vacuum degasser, thermostatic column, and refrigerated autosampler with autoinjector sample loop was used.

For sugar analysis, a ZORBAX carbohydrate analysis column (4.6 mm × 250 mm, 5 µm) (Agilent Technologies, Inc., Santa Clara, CA, USA) was applied at a temperature of 30 °C and a refractive index detector was applied. The mobile phase was a solution of acetonitrile/water (70:30, *v*/*v*) at a flow rate of 1.4 mL/min. The injection volume was 10 µL. The total analysis time was 15 min.

For AA analysis, an automatic derivatization program was set. Therefore, *o*-phthalaldehyde (OPA) and 9-fluorenylmethyl chloroformate (FMOC) reagents were used for the derivatization of primary and secondary AAs. The Agilent Zorbax Eclipse Plus C_18_ (4.6 × 150 mm, 5 µm) column (Agilent Technologies, Inc., Santa Clara, CA, USA) was used at a temperature of 40 °C to separate individual AAs. Detection of primary AAs was performed using a photodiode array detector at 388 nm, while detection of secondary AAs was performed by a fluorescence detector with an excitation wavelength of 266 nm and an emission wavelength of 305 nm. The injection volume was 5 µL and the flow rate was 1 mL/min. Eluent A of the mobile phase was 40 mM NaH_2_PO_4_ (pH 7.8, adjusted by a solution of 10 M NaOH), while eluent B was a mixture including acetonitrile/methanol/water (45:45:10, *v*/*v*/*v*). The gradient was as follows: 0–5 min, 100–90% A; 5–25 min, 90–59.5% A; 25–30 min, 59.5–37% A; 30–35 min, 37–18% A; 35–37 min, 18–0% A; 37–40 min, 0% A; and 40–43 min, 100% A.

The analytical data were integrated using Agilent OpenLab CDS ChemStation software (Agilent Technologies, Inc., Santa Clara, CA, USA) for liquid chromatography systems. Identification of sugars and AAs was performed by comparing the retention times of individual sugars and AAs in the reference solution with those in the test solution. The concentration of these compounds was assayed by comparing the peak areas obtained for the investigated samples with those of the reference solutions.

#### 4.3.3. Chemicals

The sugars and AA standards, NaOH, NaH_2_PO_4_, OPA, and FMOC were purchased from Sigma-Aldrich (St. Louis, MO, USA), while water, ACN, EtOH, and MeOH were purchased from Merck KGaA (Darmstadt, Germany).

### 4.4. Statistical Analysis

The R statistical environment was used to analyze the data and prepare the graphics [120]. The datasets for floral display, flower traits, sugars, and AAs were subjected to the Kruskal–Wallis test followed by a pairwise Wilcoxon rank sum test with Benjamini–Hochberg adjustment. The Shapiro–Wilk and Bartlett tests were used for verification as well. Furthermore, a set of descriptive statistics (mean, standard error, quartiles, and interquartile range) was calculated for the floral display, flower structure, sugars, and AAs. For all tests, the significance level was α = 0.05. To check the monotonic relationship between parameters, Spearman’s rank correlations were calculated. The correlations were considered significant for *p* < 0.05.

To analyze the effect of AAs on insect chemoreceptors, all identified and determined AAs were grouped into four taste classes: I. Asn, Gln, Ala, Cys, Gly, Ser, Thr, and Tyr (without effect on the chemoreceptors of insects); II. Arg, Asp, Glu, His, and Lys (inhibition of fly chemoreceptors); III. Pro (stimulation of the salt cell); and IV. Ile, Leu, Met, Phe, Trp, and Val (ability to stimulate the sugar cell), presented as a ternary plot [121]. The full names of the AA abbreviations are presented in the footnote to Table 3.

## 5. Conclusions

The results of our studies provide a wide view of the characteristics of flower traits (morphology and nectar) of the specialist orchid *G. repens*, which occurs in one type of habitat (coniferous forests) and is pollinated by generalist *Bombus* species. Consideration of the relationship between flower reward and pollinator requirements in the context of reproductive success enriches the existing knowledge about the evolution of plant–pollinator interactions. We found great variation in all flower traits between populations, although nectar properties had a greater impact on RS than flower morphology. The lack of correlations between flower structure and RS may indicate that *G. repens* flowers are well adapted to pollination by bumblebees. Among nectar traits, sugars play a less important role as phagostimulants compared to AAs. The nectar of *G. repens* is diluted, and is dominated by hexoses. Certain sugars parameters were correlated with PR or FRS in only three populations, although at the species level the sum of sugars, their concentration, and the amount of particular sugars positively influenced PR. However, none of these parameters were correlated with FRS. We noted the influence of 25 AAs on PR, and only four of them influenced FRS (Pro positively and Leu, Orn, and GABA negatively). Similarly, at the population level PR depended more on AAs than did FRS. The results of our studies suggest the importance of co-flowering plants for orchid RS. Because different nectar components influenced the RS parameters, and in different ways (i.e., negatively or positively), we suggest that different *Bombus* species pollinate *G. repens* flowers in distinct populations, and may play the role of main pollinators.

## Figures and Tables

**Table 1:** Floral display and flower structure in populations of *Goodyera repens*. Data represent the mean $\left(\bar{x}\right)$ ± standard deviation (SD), median $\left(\tilde{x}\right)$, interquartile range (IQR), and biological replicate (n). Different letters indicate statistically significant differences according to the pairwise Wilcoxon rank sum test with Benjamini–Hochberg adjustment (*p* < 0.05).

Statistic	Population							
	BIA1	BIA2	FORT	GRU	KRA	LIP	SOS	WIG
FLORAL DISPLAY								
Shoot height (SH) (cm)								
$\bar{x}\pm \mathrm{SD}$	19.62 ± 3.89	22.30 ± 4.08	17.0 ± 3.47	20.84 ± 3.58	19.98 ± 4.47	19.91 ± 3.87	21.96 ± 3.55	23.47 ± 4.36
$\tilde{x}$	19.25	23.0	16.50	20.0	19.0	19.50	22.50	22.50
IQR	4.62 ^a^	5.38 ^abc^	4.50 ^d^	4.0 ^abc^	6.12 ^ab^	3.88 ^a^	5.0 ^bc^	5.0 ^c^
n	20	20	31	13	30	28	39	17
Inflorescence length (IL) (cm)								
$\bar{x}\pm \mathrm{SD}$	6.42 ± 1.66	6.70 ± 1.66	7.03 ± 2.09	8.04 ± 1.71	6.55 ± 2.58	8.14 ± 2.86	6.87 ± 2.09	7.29 ± 2.05
$\tilde{x}$	6.0	6.75	6.50	8.0	5.75	7.50	6.50	7.0
IQR	2.50 ^a^	2.12 ^a^	3.0 ^a^	0.20 ^a^	2.50 ^a^	2.88 ^a^	2.50 ^a^	3.0 ^a^
n	20	20	31	13	30	28	39	17
Number of flowers (NF)								
$\bar{x}\pm \mathrm{SD}$	17.14 ± 4.36	18.25 ± 7.15	18.06 ± 4.90	18.08 ± 5.68	17.67 ± 7.25	20.46 ± 7.74	18.83 ± 5.56	17.38 ± 5.43
$\tilde{x}$	18.0	20.0	17.0	19.0	18.0	20.0	18.0	18.5
IQR	(7.0) ^a^	(9.25) ^a^	(5.5) ^a^	(5.0) ^a^	(11.75) ^a^	(10.50) ^a^	(8.0) ^a^	(4.25) ^a^
n	21	20	31	13	30	28	41	16
**FLOWER STRUCTURE**								
Flower length (FL) (mm)								
$\bar{x}\pm \mathrm{SD}$	5.26 ± 0.67	5.05 ± 0.60	5.78 ± 0.64	6.60 ± 0.61	5.83 ± 0.61	6.07 ± 0.54	5.99 ± 0.68	6.07 ± 0.83
$\tilde{x}$	5.25	5.06	5.78	6.49	5.80	6.03	6.03	6.12
IQR	0.79 ^a^	0.96 ^a^	0.80 ^b^	0.73 ^c^	1.15 ^bd^	0.75 ^d^	0.74 ^bd^	0.97 ^bcd^
n	29	22	29	11	30	32	32	21
Flower width (FW) (mm)								
$\bar{x}\pm \mathrm{SD}$	8.15 ± 1.52	7.67 ± 1.41	8.57 ± 089	9.67 ± 1.78	8.89 ± 1.09	8.99 ± 0.76	9.18 ± 0.83	9.39 ± 0.88
$\tilde{x}$	8.27	7.54	8.65	10.05	9.14	8.84	9.28	9.56
IQR	1.79 ^ab^	1.46 ^a^	1.08 ^bc^	1.09 ^d^	1.38 ^ce^	1.22 ^ce^	0.82 ^de^	1.30 ^de^
n	30	22	29	11	30	32	32	21
Length of labellum (LL) (mm)								
$\bar{x}\pm \mathrm{SD}$	2.86 ± 0.53	2.84 ± 0.37	3.32 ± 0.32	3.6 ± 0.46	3.17 ± 0.36	3.36 ± 0.28	3.37 ± 0.34	3.35 ± 0.28
$\tilde{x}$	2.96	2.86	3.29	3.59	3.15	3.37	3.38	3.34
IQR	0.66 ^a^	0.58 ^a^	0.44 ^bc^	0.43 ^b^	0.55 ^c^	0.43 ^bc^	0.49 ^bc^	0.33 ^bc^
n	30	22	29	11	30	32	32	21
Width of labellum (WL) (mm)								
$\bar{x}\pm \mathrm{SD}$	1.37 ± 0.51	1.24 ± 0.31	1.64 ± 0.24	1.57 ± 0.23	1.48 ± 0.25	1.64 ± 0.28	1.54 ± 0.25	1.64 ± 0.26
$\tilde{x}$	1.31	1.23	1.64	1.50	1.45	1.70	1.54	1.67
IQR	0.43 ^ab^	0.45 ^a^	0.25 ^c^	0.18 ^bcd^	0.34 ^bd^	0.41 ^cd^	0.34 ^cd^	0.36 ^cd^
n	30	22	29	11	30	32	32	21
Length of hypochile (LH) (mm)								
$\bar{x}\pm \mathrm{SD}$	1.32 ± 0.32	1.21 ± 0.21	1.45 ± 0.22	1.47 ± 0.20	1.26 ± 0.19	1.39 ± 0.16	1.35 ± 0.17	1.44 ± 0.15
$\tilde{x}$	1.34	1.28	1.42	1.45	1.25	1.40	1.41	1.44
IQR	0.38 ^abc^	0.24 ^a^	0.29 ^b^	0.21 ^b^	0.27 ^ac^	0.21 ^b^	0.30 ^bc^	0.18 ^b^
n	29	22	29	11	30	32	32	21

**Table 2:** Ratios of sugars in *Goodyera repens* nectar. Data represent the mean $\left(\bar{x}\right)$ ± standard deviation (SD), median $\left(\tilde{x}\right)$, interquartile range (IQR), and biological replicate (n). Different letters indicate statistically significant differences according to the pairwise Wilcoxon rank sum test with Benjamini–Hochberg adjustment (*p* < 0.05).

Statistic	Population							
	BIA1	BIA2	FORT	GRU	KRA	LIP	SOS	WIG
n	30	22	30	11	30	28	41	21
Sucrose/Fructose + Glucose								
$\bar{x}\pm \mathrm{SD}$	0.25 ± 0.03	0.23 ± 0.02	0.22 ± 0.04	0.44 ± 0.05	0.18 ± 0.03	0.21 ± 0.03	0.25 ± 0.03	0.28 ± 0.04
$\tilde{x}$	0.24	0.22	0.22	0.44	0.18	0.21	0.25	0.28
IQR	0.04 ^a^	0.03 ^b^	0.05 ^bc^	0.07 ^d^	0.05 ^e^	0.04 ^c^	0.05 ^a^	0.04 ^f^
Glucose/Fructose								
$\bar{x}\pm \mathrm{SD}$	1.03 ± 0.07	1.05 ± 0.09	0.99 ± 0.09	1.04 ± 0.10	1.35 ± 0.12	0.98 ± 0.07	1.0 ± 0.11	0.71 ± 0.08
$\tilde{x}$	1.02	1.03	0.97	1.0	1.34	0.99	1.03	0.71
IQR	0.09 ^ab^	0.08 ^a^	0.14 ^b^	0.08 ^ab^	0.10 ^c^	0.09 ^b^	0.12 ^ab^	0.11 ^d^

**Table 3:** Amino acid concentration (µM) in *Goodyera repens* nectar. Data represent the mean $\left(\bar{x}\right)$ ± standard deviation (SD), median $\left(\tilde{x}\right)$, interquartile range (IQR), and biological replicate (n). Different letters indicate statistically significant differences according to the pairwise Wilcoxon rank sum test with Benjamini–Hochberg adjustment (*p* < 0.05).

Amino Acid	Statistic	Population							
		BIA1	BIA2	FORT	GRU	KRA	LIP	SOS	WIG
		n = 30	n = 22	n = 30	n = 11	n = 30	n = 28	n = 41	n = 21
Proteogenic AAs									
Asp nEAA, I	$\bar{x}\pm \mathrm{SD}$	58.66 ± 7.12	68.37 ± 5.73	97.85 ± 16.01	119.15 ± 18.36	94.99 ± 14.29	52.65 ± 11.45	63.79 ± 10.23	26.72 ± 4.80
	$\tilde{x}$	56.85	68.28	97.43	124.4	93.53	52.39	61.79	25.99
	IQR	11.30 ^a^	6.76 ^b^	23.61 ^c^	25.47 ^d^	21.77 ^c^	11.62 ^e^	10.40 ^f^	4.89 ^g^
Glu nEAA, I	$\bar{x}\pm \mathrm{SD}$	60.60 ± 7.93	113.34 ± 7.27	76.48 ± 13.38	32.50 ± 8.73	97.36 ± 16.49	13.24 ± 2.12	11.40 ± 1.89	8.0 ± 1.37
	$\tilde{x}$	59.35	112.68	76.94	29.68	95.60	13.63	11.35	7.62
	IQR	11.04 ^a^	9.67 ^b^	14.29 ^c^	9.62 ^d^	23.03 ^e^	3.0 ^f^	3.0 ^g^	1.26 ^h^
Ala nEAA, I	$\bar{x}\pm \mathrm{SD}$	45.36 ± 4.99	130.03 ± 6.57	29.09 ± 3.97	12.02 ± 2.31	42.88 ± 7.31	15.05 ± 2.09	13.93 ± 1.62	21.05 ± 2.62
	$\tilde{x}$	46.94	128.96	28.70	11.60	40.91	15.95	13.67	20.51
	IQR	10.30 ^a^	7.10 ^b^	5.58 ^c^	3.19 ^d^	8.96 ^a^	2.64 ^e^	1.80 ^f^	3.41 ^g^
Cys nEAA, I	$\bar{x}\pm \mathrm{SD}$	12.03 ± 0.48	20.11 ± 2.45	13.93 ± 2.22	8.59 ± 1.37	23.99 ± 3.37	8.48 ± 1.39	5.12 ± 0.75	17.02 ± 2.16
	$\tilde{x}$	12.05	19.76	14.06	8.33	23.16	8.80	5.11	15.83
	IQR	0.42 ^a^	3.80 ^b^	3.51 ^c^	1.63 ^d^	4.30 ^e^	1.50 ^d^	1.11 ^f^	2.77 ^g^
Gly nEAA, I	$\bar{x}\pm \mathrm{SD}$	23.75 ± 2.44	71.68 ± 3.15	51.97 ± 8.57	41.08 ± 6.43	46.10 ± 6.58	19.69 ± 3.09	46.52 ± 5.30	29.21 ± 6.62
	$\tilde{x}$	24.13	70.98	52.30	41.74	45.02	19.95	46.65	28.95
	IQR	4.17 ^a^	5.43 ^b^	11.55 ^c^	6.97 ^d^	8.04 ^e^	4.42 ^f^	9.07 ^e^	7.65 ^g^
Ser nEAA, I	$\bar{x}\pm \mathrm{SD}$	82.47 ± 4.83	153.84 ± 13.08	64.34 ± 9.23	64.81 ± 11.0	77.52 ± 11.69	10.02 ± 1.40	10.02 ± 1.21	5.33 ± 1.78
	$\tilde{x}$	82.45	154.94	64.71	67.31	72.10	10.25	10.22	5.09
	IQR	6.30 ^a^	22.69 ^b^	14.23 ^c^	13.39 ^c^	17.63 ^d^	1.48 ^e^	2.24 ^e^	1.36 ^f^
Thr EAA, I	$\bar{x}\pm \mathrm{SD}$	10.96 ± 0.49	11.57 ± 1.13	7.66 ± 1.16	8.75 ± 1.54	19.61 ± 3.82	8.24 ± 1.43	8.38 ± 0.94	15.96 ± 2.11
	$\tilde{x}$	10.94	11.32	7.54	8.35	18.59	8.12	8.56	15.06
	IQR	0.71 ^a^	2.02 ^a^	1.08 ^b^	1.79 ^c^	4.84 ^d^	1.90 ^bc^	1.43 ^c^	3.11 ^e^
Tyr nEAA, I	$\bar{x}\pm \mathrm{SD}$	0.51 ± 0.31	8.67 ± 0.47	5.25 ± 0.73	5.09 ± 0.87	6.13 ± 0.91	6.46 ± 0.88	2.41 ± 0.34	3.14 ± 0.39
	$\tilde{x}$	0.62	8.68	5.22	5.02	6.02	6.52	2.41	3.09
	IQR	0.30 ^a^	0.81 ^b^	0.91 ^c^	0.82 ^c^	1.31 ^d^	1.06 ^d^	0.44 ^e^	0.48 ^f^
Arg EAA, I	$\bar{x}\pm \mathrm{SD}$	8.04 ± 0.60	22.27 ± 1.0	10.96 ± 1.76	11.58 ± 1.24	22.09 ± 3.13	8.69 ± 1.46	9.91 ± 1.07	21.52 ± 2.87
	$\tilde{x}$	7.94	22.01	10.73	11.42	22.29	8.56	9.86	20.73
	IQR	0.80 ^a^	1.37 ^b^	2.49 ^c^	1.74 ^c^	4.10 ^b^	1.84 ^d^	1.49 ^e^	3.92 ^b^
Asn nEAA, I	$\bar{x}\pm \mathrm{SD}$	65.67 ± 3.71	117.69 ± 8.44	60.68 ± 7.55	7.78 ± 1.29	90.26 ± 12.13	5.95 ± 1.0	6.80 ± 0.96	13.43 ± 1.88
	$\tilde{x}$	64.22	118.54	59.86	7.73	89.03	6.02	6.89	13.17
	IQR	5.16 ^a^	10.55 ^b^	12.67 ^c^	1.34 ^d^	17.67 ^e^	1.45 ^f^	1.36 ^g^	2.28 ^h^
Gln nEAA, I	$\bar{x}\pm \mathrm{SD}$	61.20 ± 4.39	68.71 ± 5.10	49.05 ± 8.16	82.14 ± 12.58	60.33 ± 8.88	8.07 ± 1.30	14.59 ± 1.48	26.63 ± 3.70
	$\tilde{x}$	62.06	68.14	49.77	83.39	57.98	8.32	15.11	25.98
	IQR	6.27 ^a^	6.33 ^b^	12.20 ^c^	15.97 ^d^	11.52 ^a^	1.74 ^e^	2.47 ^f^	4.02 ^g^
His EAA, II	$\bar{x}\pm \mathrm{SD}$	11.77 ± 0.76	11.75 ± 1.12	39.34 ± 5.39	39.02 ± 6.02	32.69 ± 5.37	7.18 ± 1.21	17.60 ± 2.09	36.55 ± 5.96
	$\tilde{x}$	11.70	11.78	39.64	37.82	32.28	7.16	17.62	35.78
	IQR	1.14 ^a^	1.36 ^a^	6.93 ^b^	4.28 ^b^	6.63 ^c^	1.47 ^d^	3.62 ^e^	8.80 ^b^
Lys EAA, II	$\bar{x}\pm \mathrm{SD}$	4.07 ± 0.41	12.45 ± 0.80	12.51 ± 1.82	4.53 ± 0.77	27.14 ± 4.07	4.74 ± 0.99	5.91 ± 0.74	8.43 ± 1.10
	$\tilde{x}$	3.98	12.32	12.32	4.76	26.24	4.58	5.85	8.31
	IQR	0.67 ^a^	1.31 ^b^	2.64 ^b^	0.87 ^c^	6.80 ^d^	1.28 ^c^	0.99 ^e^	1.72 ^f^
Pro nEAA, III	$\bar{x}\pm \mathrm{SD}$	20.16 ± 0.81	22.81 ± 1.18	11.79 ± 1.87	28.0 ± 4.11	28.84 ± 3.78	11.50 ± 1.85	15.80 ± 2.10	9.32 ± 1.25
	$\tilde{x}$	20.12	22.79	11.89	26.42	28.59	11.66	15.51	9.02
	IQR	1.55 ^a^	0.98 ^b^	2.75 ^c^	4.14 ^d^	5.59 ^d^	2.81 ^c^	3.11 ^e^	2.01 ^f^
Ile EAA, IV	$\bar{x}\pm \mathrm{SD}$	13.15 ± 0.62	22.75 ± 2.68	11.38 ± 1.74	6.86 ± 1.29	18.0 ± 2.67	7.85 ± 1.17	7.04 ± 0.92	11.20 ± 1.51
	$\tilde{x}$	13.21	23.22	11.72	6.62	17.04	7.84	6.87	10.93
	IQR	0.85 ^a^	4.85 ^b^	2.11 ^c^	0.96 ^d^	3.94 ^e^	1.92 ^f^	1.34 ^d^	1.76 ^c^
Leu EAA, IV	$\bar{x}\pm \mathrm{SD}$	9.42 ± 0.86	25.95 ± 1.83	28.53 ± 4.13	9.12 ± 1.48	26.64 ± 4.39	7.28 ± 1.30	9.78 ± 1.05	5.51 ± 0.90
	$\tilde{x}$	9.51	26.01	28.43	9.02	26.16	7.0	9.69	5.33
	IQR	1.11 ^a^	3.28 ^b^	5.91 ^c^	1.61 ^a^	7.10 ^b^	2.04 ^d^	1.20 ^a^	1.37 ^e^
Met EAA, IV	$\bar{x}\pm \mathrm{SD}$	2.51 ± 0.37	4.24 ± 0.48	2.67 ± 0.44	0.66 ± 0.46	6.5 ± 0.98	0.75 ± 0.27	1.18 ± 0.25	2.46 ± 0.41
	$\tilde{x}$	2.51	4.15	2.76	0.82	6.42	0.80	1.16	2.46
	IQR	0.55 ^a^	0.81 ^b^	0.49 ^a^	0.60 ^c^	1.50 ^d^	0.24 ^c^	0.28 ^e^	0.28 ^a^
Phe EAA, IV	$\bar{x}\pm \mathrm{SD}$	15.17 ± 0.53	10.71 ± 0.94	10.40 ± 1.25	9.42 ± 1.62	18.45 ± 2.87	6.05 ± 0.90	8.17 ± 0.90	15.35 ± 1.86
	$\tilde{x}$	15.22	10.36	10.53	9.62	17.86	6.15	8.18	14.82
	IQR	0.75 ^a^	1.22 ^b^	1.49 ^b^	1.79 ^c^	3.92 ^d^	1.24 ^e^	1.51 ^f^	2.02 ^a^
Trp EAA, IV	$\bar{x}\pm \mathrm{SD}$	22.86 ± 1.87	40.47 ± 2.42	10.02 ± 1.83	11.82 ± 2.17	19.37 ± 3.02	8.63 ± 1.27	8.61 ± 1.03	12.59 ± 1.56
	$\tilde{x}$	23.69	40.77	9.93	11.08	18.87	8.52	8.69	12.07
	IQR	2.81 ^a^	2.17 ^b^	2.31 ^c^	3.24 ^d^	3.96 ^e^	1.03 ^f^	1.29 ^f^	1.92 ^d^
Val EAA, IV	$\bar{x}\pm \mathrm{SD}$	18.62 ± 0.53	12.38 ± 0.37	10.81 ± 1.51	8.02 ± 1.52	15.87 ± 2.27	11.86 ± 1.98	4.17 ± 0.69	15.62 ± 2.01
	$\tilde{x}$	18.73	12.37	10.73	8.0	15.56	12.01	4.11	14.79
	IQR	0.77 ^a^	0.49 ^b^	2.32 ^c^	1.04 ^d^	2.37 ^e^	2.43 ^b^	1.19 ^f^	2.36 ^e^
Sum of proteogenic AAs	$\bar{x}\pm \mathrm{SD}$	546.95 ± 11.22	949.79 ± 30.46	604.69 ± 70.22	510.93 ± 80.35	774.74 ± 108.19	222.38 ± 32.73	271.12 ± 26.93	305.04 ± 40.82
	$\tilde{x}$	549.60	948.58	611.05	540.84	729.71	232.37	279.51	284.67
	IQR	12.32 ^a^	37.68 ^b^	111.24 ^c^	96.30 ^d^	157.27 ^e^	39.97 ^f^	42.86 ^g^	50.77 ^h^
**Non-proteogenic AAs**									
Orn	$\bar{x}\pm \mathrm{SD}$			5.26 ± 0.78	3.85 ± 0.61	3.60 ± 0.72	2.77 ± 0.43	3.68 ± 0.59	4.87 ± 0.91
	$\tilde{x}$			5.26	3.89	3.51	2.79	3.71	4.82
	IQR			0.82 ^a^	0.95 ^b^	0.81 ^b^	0.60 ^c^	0.62 ^b^	0.78 ^a^
Cit	$\bar{x}\pm \mathrm{SD}$	3.48 ± 0.50	1.72 ± 0.15	1.47 ± 0.23	2.98 ± 0.43	2.62 ± 0.37		0.94 ± 0.35	
	$\tilde{x}$	3.60	1.79	1.50	2.82	2.66		1.0	
	IQR	0.79 ^a^	0.20 ^b^	0.38 ^c^	0.56 ^d^	0.49 ^e^		0.31 ^f^	
Tau	$\bar{x}\pm \mathrm{SD}$	4.72 ± 0.44	11.11 ± 0.98	2.82 ± 0.47	3.50 ± 0.68	8.25 ± 1.17	9.0 ± 1.36	6.16 ± 0.76	6.18 ± 0.90
	$\tilde{x}$	4.72	11.03	2.77	3.40	7.92	8.75	6.22	6.0
	IQR	0.58 ^a^	1.55 ^b^	0.68 ^c^	0.68 ^d^	1.77 ^e^	1.90 ^f^	1.18 ^g^	0.74 ^g^
AABA	$\bar{x}\pm \mathrm{SD}$	2.98 ± 0.30	4.72 ± 0.34	2.86 ± 0.46		2.91 ± 0.50	2.41 ± 0.45	2.63 ± 0.36	1.59 ± 0.24
	$\tilde{x}$	2.96	4.66	2.85		2.87	2.44	2.60	1.63
	IQR	0.45 ^a^	0.55 ^b^	0.62 ^a^		0.85 ^a^	0.64 ^c^	0.41 ^d^	0.32 ^e^
BABA	$\bar{x}\pm \mathrm{SD}$	8.10 ± 0.67	8.15 ± 0.56	7.32 ± 1.13	2.99 ± 0.61	9.62 ± 1.21			
	$\tilde{x}$	8.05	8.10	7.42	2.99	9.60			
	IQR	1.07 ^a^	0.82 ^a^	1.69 ^b^	0.99 ^c^	2.03 ^d^			
GABA	$\bar{x}\pm \mathrm{SD}$	12.20 ± 0.83	13.81 ± 2.21	17.56 ± 2.20	8.0 ± 1.14	18.51 ± 2.60	6.24 ± 0.93	6.45 ± 0.82	4.69 ± 0.48
	$\tilde{x}$	12.02	13.13	17.90	7.81	17.65	6.31	6.51	4.78
	IQR	1.32 ^a^	3.43 ^b^	3.02 ^c^	1.18 ^d^	3.46 ^c^	0.99 ^e^	1.13 ^e^	0.60 ^f^
Sum of non-proteogenic AAs	$\bar{x}\pm \mathrm{SD}$	31.47 ± 1.56	39.50 ± 2.92	37.30 ± 4.53	21.32 ± 2.82	45.51 ± 6.13	20.42 ± 2.87	19.86 ± 2.11	17.32 ± 2.15
	$\tilde{x}$	31.55	39.17	36.97	20.89	42.70	20.41	20.02	17.19
	IQR	2.29 ^a^	4.78 ^b^	7.04 ^b^	3.49 ^c^	9.31 ^d^	3.97 ^c^	3.18 ^c^	2.51 ^e^

AABA, α-aminobutyric acid; Ala, L-alanine; Arg, L-arginine; Asn, L-asparagine; Asp, L-aspartic acid; BABA, β-aminobutyric acid; Cit, L-citrulline; Cys, L-cystine; GABA, γ-aminobutyric acid; Gln, L-glutamine; Glu, L-glutamic acid; Gly, glycine; His, L-histidine; Ile, L-isoleucine; Leu, L-leucine; Lys, L-lysine; Met, L-methionine; Orn, L-ornithine; Phe, L-phenylalanine; Pro, L-proline; Ser, L-serine; Tau, taurine; Thr, L-threonine; Trp, L-tryptophan; Tyr, L-tyrosine; Val, L-valine; EAA, essential amino acid; nEAA, non-essential amino acid. The number of classes represents the effect of amino acids on insect chemoreceptors: I, no effect; II, inhibition of chemoreceptors; III, stimulation of the salt cell; IV, the ability to stimulate the sugar cell.

**Table 4:** Spatial variation of pollinaria removal (PR) and female reproductive success (FRS) in populations of *Goodyera repens.* Data represent the mean $\left(\bar{x}\right)$ ± standard deviation (SD), median $\left(\tilde{x}\right)$, interquartile range (IQR), and biological replicate (n). Different letters indicate statistically significant differences according to the pairwise Wilcoxon rank sum test with Benjamini–Hochberg adjustment (*p* < 0.05).

Statistic	Population							
	BIA1	BIA2	FORT	GRU	KRA	LIP	SOS	WIG
PR (%)								
$\bar{x}\pm \mathrm{SD}$			75.04 ± 26.47	76.68 ± 20.18	78.64 ± 21.38	67.65 ± 27.81	51.60 ± 25.58	78.13 ± 28.26
$\tilde{x}$			83.33	81.07	81.81	78.02	52.63	83.33
IQR			32.47 ^a^	22.91 ^a^	25.25 ^a^	33.43 ^a^	31.89 ^b^	22.22 ^a^
n			31	12	27	28	39	17
FRS (%)								
$\bar{x}\pm \mathrm{SD}$	76.10 ± 23.29	78.06 ± 17.82	56.12 ± 29.26	77.77 ± 12.12	74.18 ± 21.70	66.06 ± 30.13	76.27 ± 22.98	65.15 ± 37.96
$\tilde{x}$	80.47	77.50	59.10	75.25	71.43	80.0	80.0	78.57
IQR	19.87 ^ab^	27.25 ^ab^	34.46 ^a^	13.51 ^ab^	33.63 ^ab^	39.83 ^ab^	20.28 ^b^	30.91 ^ab^
n	20	20	31	12	27	28	39	17
PR/FRS								
$\bar{x}\pm \mathrm{SD}$			1.53 ± 1.30	1.0 ± 0.30	1.13 ± 0.37	3.98 ± 14.58	1.06 ± 2.17	1.48 ± 1.60
$\tilde{x}$			1.12	1.0	1.11	1.0	0.72	1.02
IQR			0.87 ^a^	0.37 ^ab^	0.39 ^a^	0.73 ^a^	0.52 ^b^	0.25 ^a^
n			28	12	27	28	39	14

**Table 5:** Impact of floral display, flower structures, and nectar composition on the reproductive success in *Goodyera repens* populations, expressed as pollinaria removal (PR) and female reproductive success (fruiting, FRS).

Population	Floral Display	Flower Structure	Sugars	Amino Acids
BIA1	SH → FRS (r_S_ = 0.57)	FL → FRS (r_S_ = 0.47)		Tyr → FRS (r_S_ = 0.45)
	IL → FRS (r_S_ = 0.49)			Tau → FRS (r_S_ = −0.46)
				Total AAs → FRS (r_S_ = 0.46)
				Taste I AAs → FRS (r_S_ = 0.46)
				nEAAs → FRS (r_S_ = −0.63)
BIA2	IL → FRS (r_S_ = 0.47)			Ile → FRS (r_S_ = −0.74)
				Tau → FRS (r_S_ = −0.51)
FORT	IL → FRS (r_S_ = 0.37)			Ser → PR (r_S_ = 0.40)
				Lys → PR (r_S_ = 0.42)
				Leu → PR (r_S_ = 0.41)
				Non-protAAs → PR (r_S_ = 0.39)
				Taste I AAs → PR (r_S_ = 0.42)
				Orn → FRS (r_S_ = −0.57)
				Tau → FRS (r_S_ = −0.39)
				Non-protAAs → FRS (r_S_ = −0.42)
GRU			Fructose → FRS (r_S_ = −1)	Ser → PR (r_S_ = −0.88)
			Sugars → FRS (r_S_ = −0.83)	Phe → PR (r_S_ = −0.88)
				Taste IV AAs → PR (r_S_ = −0.88)
				Ile → FRS (r_S_ = −0.89)
				Val → FRS (r_S_ = −0.94)
				Taste I AAs → FRS (r_S_ = −0.89)
KRA			Sugars → PR (r_S_ = 0.43)	Asp → PR (r_S_ = 0.53)
				Glu → PR (r_S_ = 0.49)
				Thr → PR (r_S_ = 0.53)
				Arg → PR (r_S_ = 0.53)
				Asn → PR (r_S_ = 0.49)
				Gln → PR (r_S_ = 0.49)
				His → PR (r_S_ = 0.55)
				Lys → PR (r_S_ = 0.48)
				Pro → PR (r_S_ = 0.47)
				Cit → PR (r_S_ = 0.58)
				Tau → PR (r_S_ = 0.45)
				BABA → PR (r_S_ = 0.47)
				GABA → PR (r_S_ = 0.44)
				protAAs → PR (r_S_ = 0.54)
				non-protAAs → PR (r_S_ = 0.51)
				total AAs → PR (r_S_ = 0.53)
				Taste I AAs → PR (r_S_ = 0.47)
				Taste II AAs → PR (r_S_ = 0.55)
				Taste III AAs → PR (r_S_ = 0.47)
				EAAs → PR (r_S_ = 0.56)
				nEAAs → PR (r_S_ = 0.52)
LIP	IL → FRS (r_S_ = 0.37)			Asn → FRS (r_S_ = −0.48)
SOS	NF → FRS (r_S_ = −0.35)			
WIG			Fructose → PR (r_S_ = −0.62)	

Abbreviations for floral display, flower structure, and amino acids are presented in Table 1 and Table 3, respectively.

## Data Availability

Data are contained within the current article and Supplementary Materials.

## Supplementary Materials

The following supporting information can be downloaded at: https://www.mdpi.com/article/10.3390/ijms24108602/s1.
