# Supplementary material for: In Which Way Do the Flower Properties of the Specialist Orchid Goodyera repens Meet the Requirements of Its Generalist Pollinators?
Source: Int J Mol Sci. 2023 May 11;24(10):8602. doi: 10.3390/ijms24108602 (PMC10217979; doi:10.3390/ijms24108602)
Supplement: Supplementary file 1 [file ijms-24-08602-s001.zip › ijms-2381515-supplementary.pdf]

Supplementary Materials

In Which Way Do the Flower Properties of the Specialist Orchid *Goodyera repens* Meet the Requirements of Its Generalist Pollinators?

Emilia Brzosko *, Andrzej Bajguz *, Justyna Burzyńska and Magdalena Chmur

Department of Biology and Plant Ecology, Faculty of Biology, University of Bialystok, Ciolkowskiego 1J, 15-245 Bialystok, Poland; j.burzynska@uwb.edu.pl (J.B.); m.chmur@uwb.edu.pl (M.C.)
*Correspondence: emilka@uwb.edu.pl (E.B.); abajguz@uwb.edu.pl (A.B.)

Figure S1. Correlation heatmap for reproductive success (that is, pollinaria removal, PR, and female re-productive success ,FRS), floral display (that is, SH, IL, and NF), flower structures (that is, FL, FW, LL, WL, and LH), and nectar composition (that is, sugars and amino acids) of *Goodyera repens* populations. Significant positive correlations are colored blue, while negative correlations are colored red (p<0.05). Insignificant correlations are omitted. A. The colored and labeled scale codes show the value of the Spearman rank correlation coefficient (r_S). The abbreviations are explained in Tables 1 and 3.

BIA1

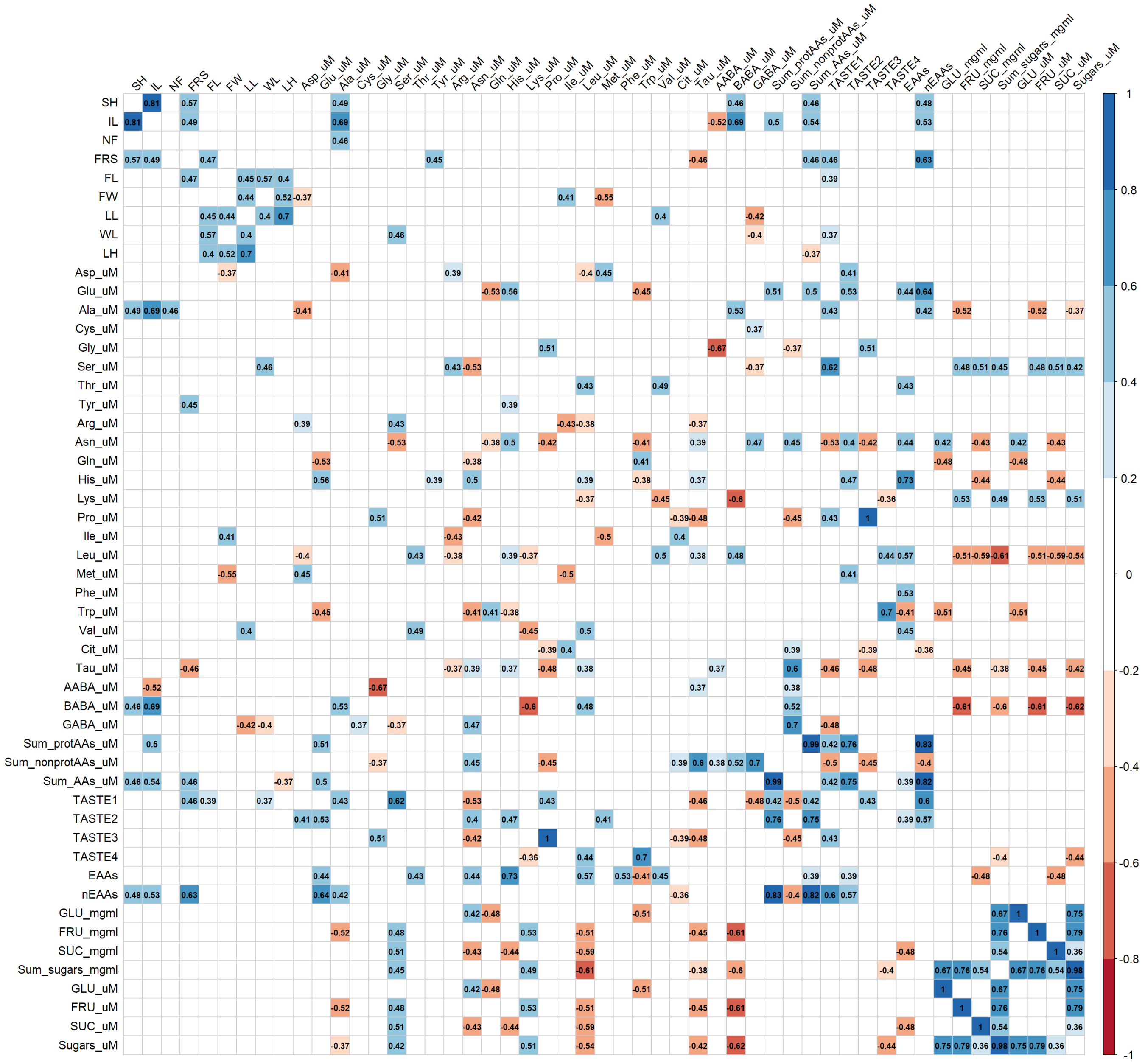

BIA2

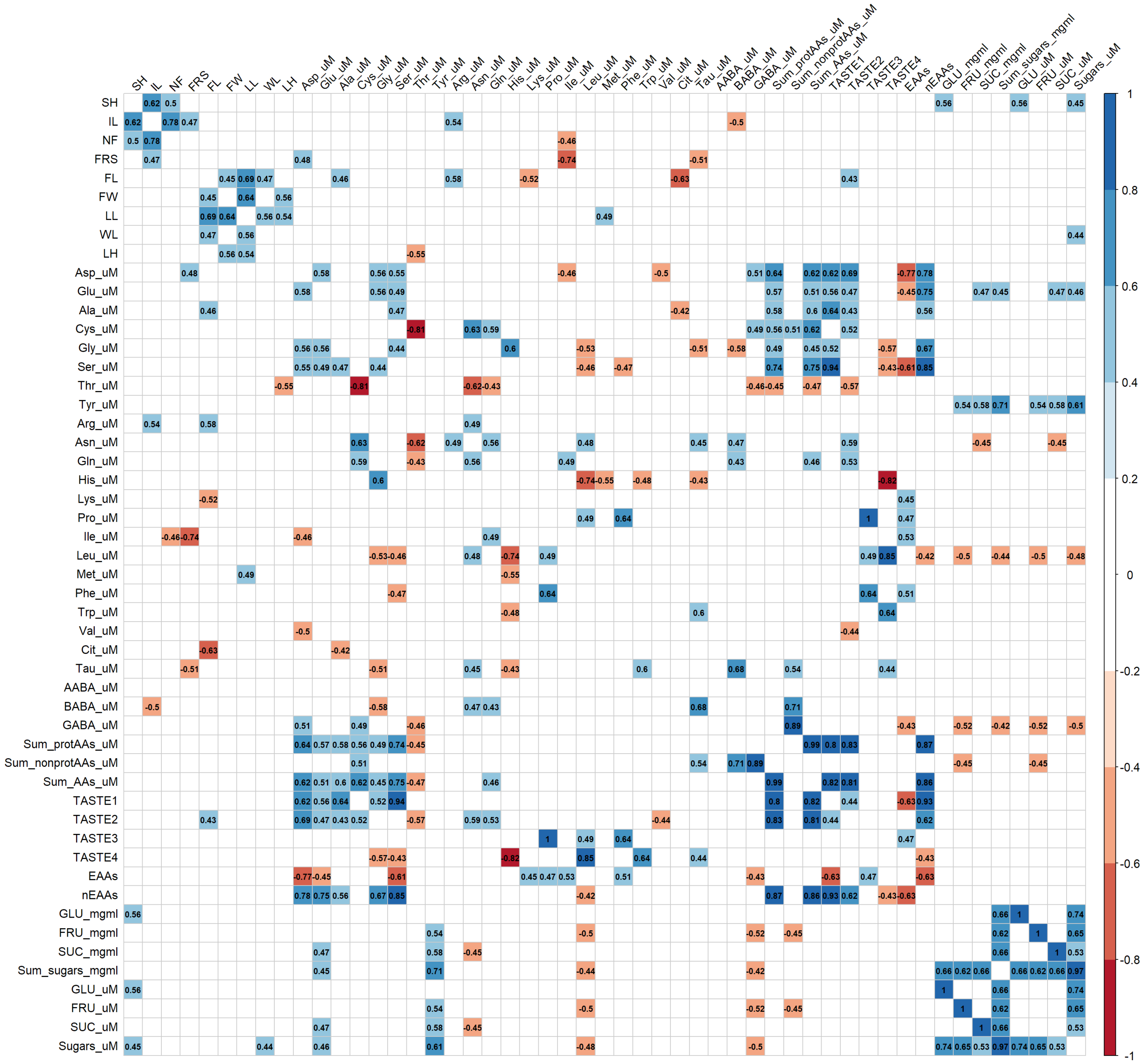
SH
IL
NF
FRS
FL
FW
LL
WL
LH
Asp_uM
Glu_uM
Ala_uM
Cys_uM
Gly_uM
Ser_uM
Thr_uM
Tyr_uM
Arg_uM
Asn_uM
Gln_uM
His_uM
Lys_uM
Pro_uM
Ile_uM
Leu_uM
Met_uM
Phe_uM
Trp_uM
Val_uM
Cit_uM
Tau_uM
AABA_uM
BABA_uM
GABA_uM
Sum_protAAs_uM
Sum_nonprotAAs_uM
Sum_AAs_uM
TASTE1
TASTE2
TASTE3
TASTE4
EAAs
nEAAs
GLU_mgml
FRU_mgml
SUC_mgml
Sum_sugars_mgml
GLU_uM
FRU_uM
SUC_uM
Sugars_uM
1
0.8
0.6
0.4
0.2
0
-0.2
-0.4
-0.6
-0.8
-1

FORT

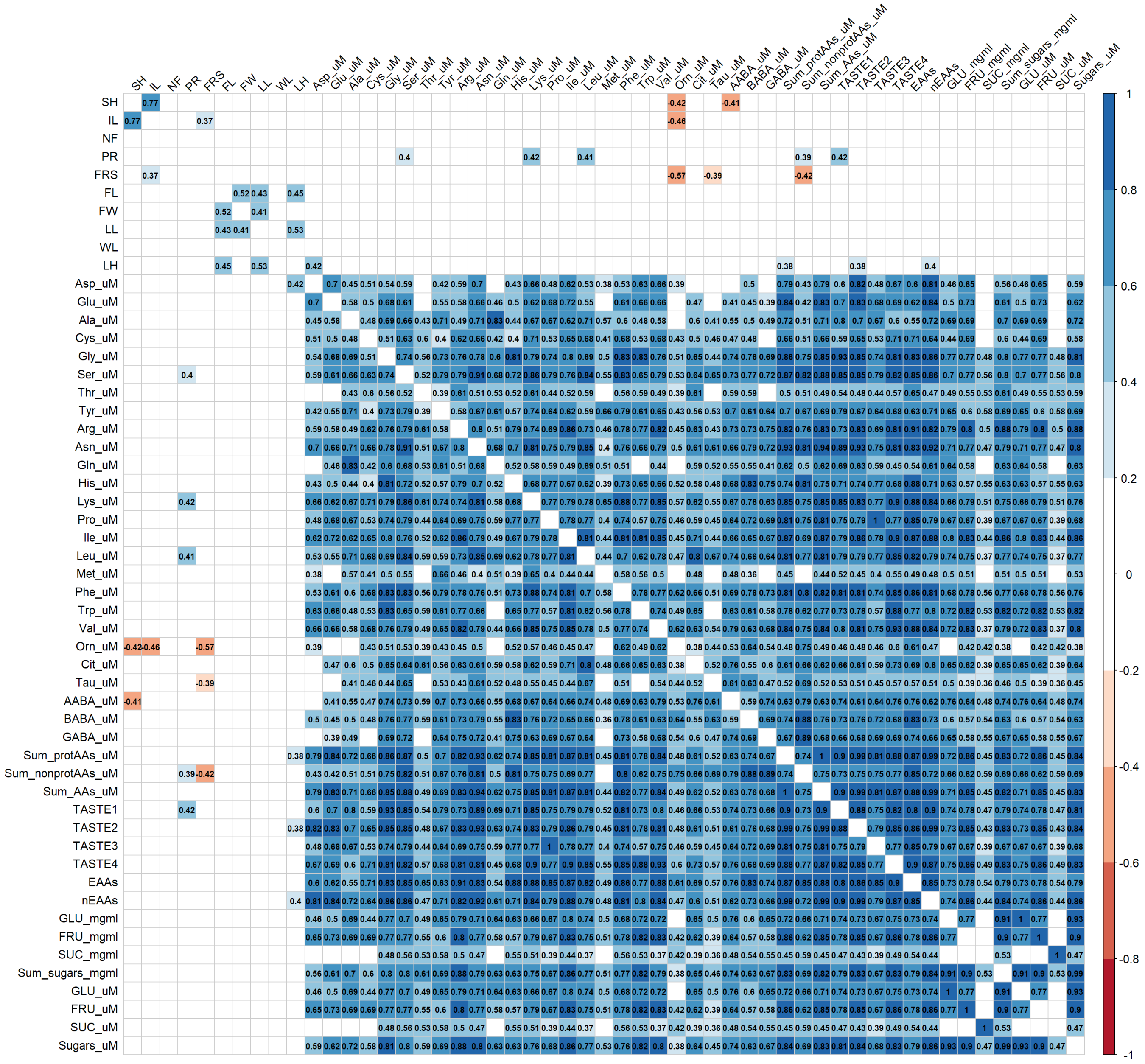
SH
IL
NF
PR
FRS
FL
FW
LL
WL
LH
Asp_uM
Glu_uM
Ala_uM
Cys_uM
Gly_uM
Ser_uM
Thr_uM
Tyr_uM
Arg_uM
Asn_uM
Gln_uM
His_uM
Lys_uM
Pro_uM
Ile_uM
Leu_uM
Met_uM
Phe_uM
Trp_uM
Val_uM
Orn_uM
Cit_uM
Tau_uM
AABA_uM
BABA_uM
GABA_uM
Sum_protAAs_uM
Sum_nonprotAAs_uM
Sum_AAs_uM
TASTE1
TASTE2
TASTE3
TASTE4
EAAs
nEAAs
GLU_mgml
FRU_mgml
SUC_mgml
Sum_sugars_mgml
GLU_uM
FRU_uM
SUC_uM
Sugars_uM
1
0.8
0.6
0.4
0.2
0
-0.2
-0.4
-0.6
-0.8
-1

GRU

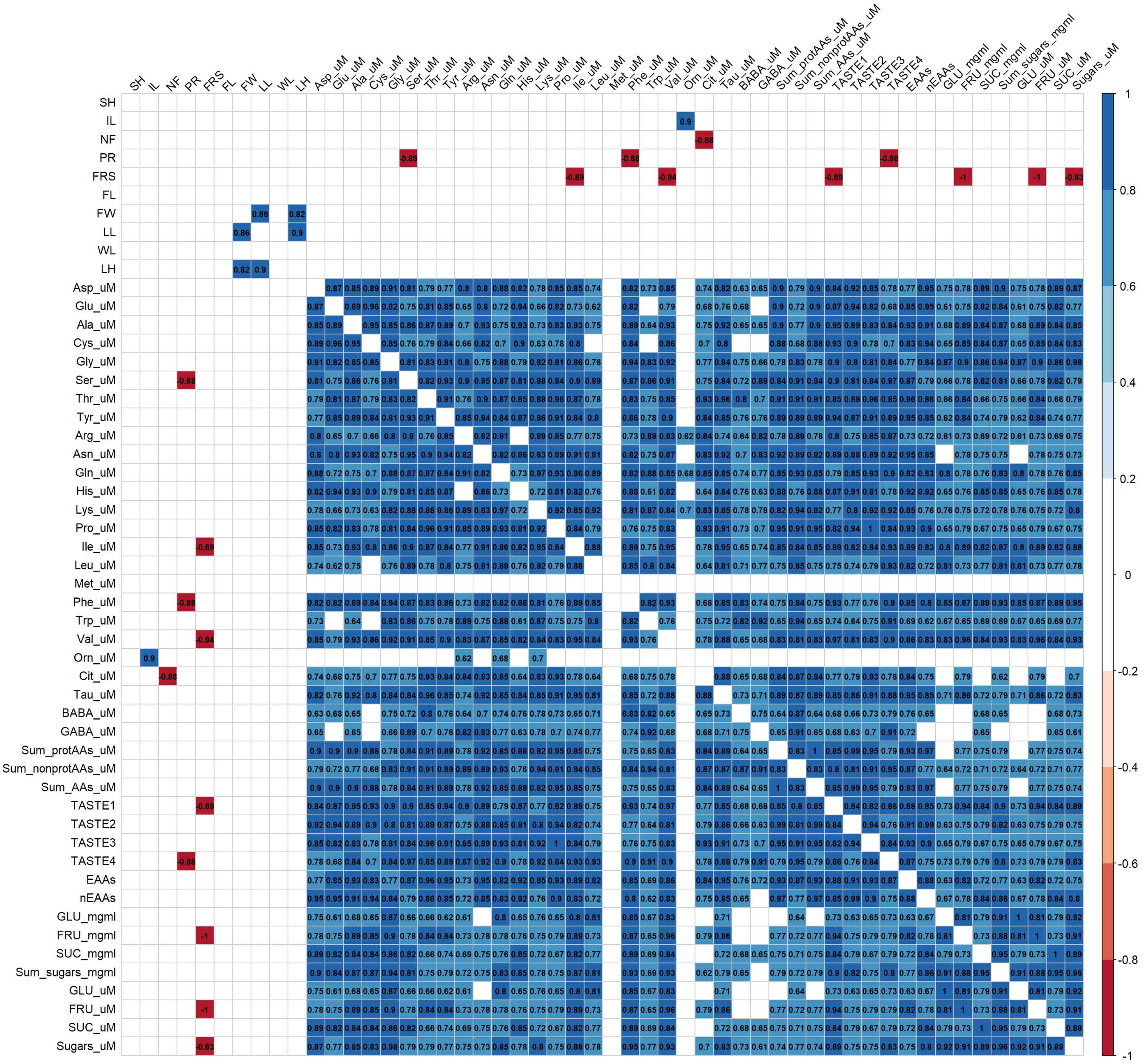

KRA

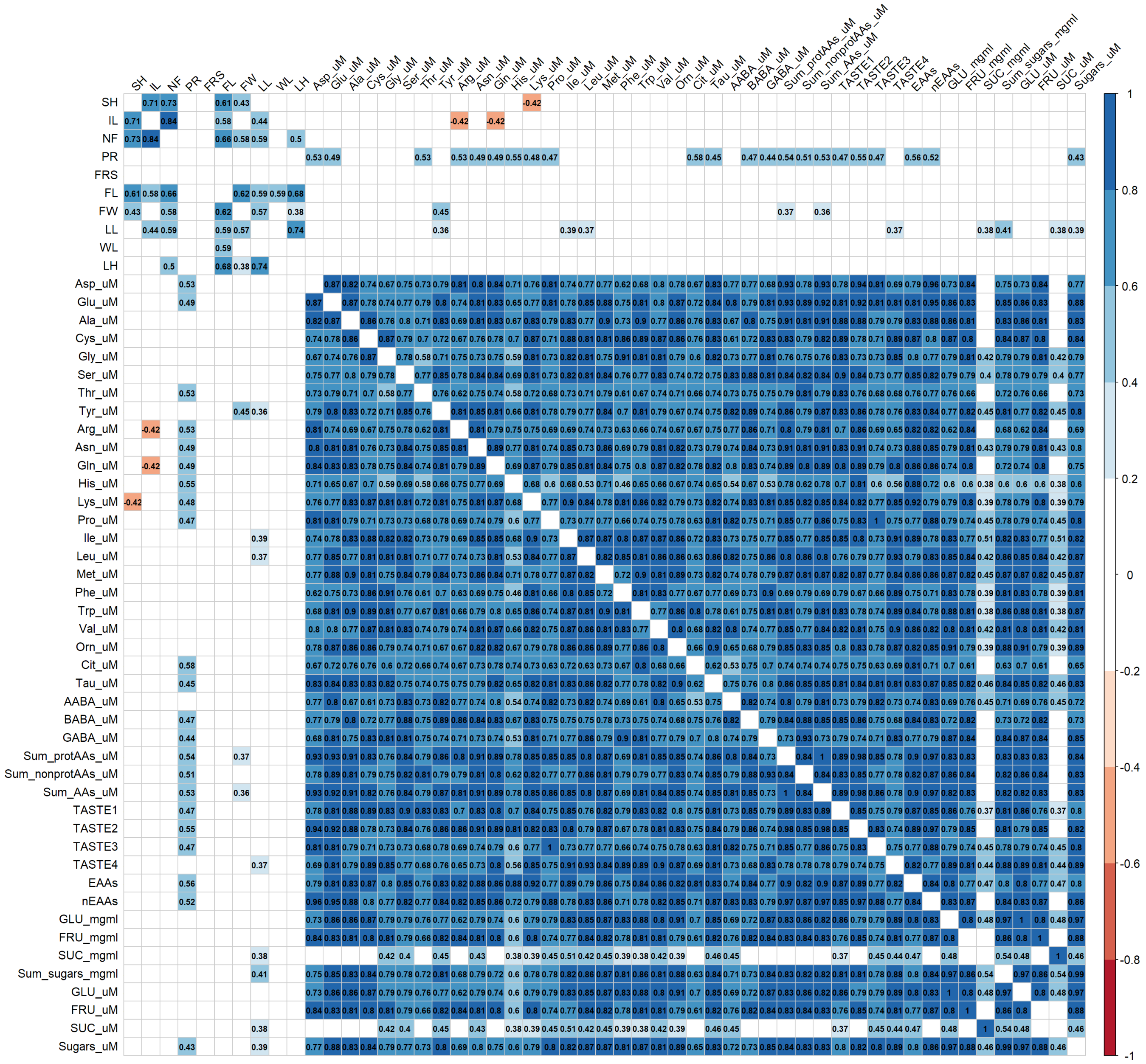

SH
IL
NF
PR
FRS
FL
FW
LL
WL
LH
Asp_uM
Glu_uM
Ala_uM
Cys_uM
Gly_uM
Ser_uM
Thr_uM
Tyr_uM
Arg_uM
Asn_uM
Gln_uM
His_uM
Lys_uM
Pro_uM
Ile_uM
Leu_uM
Met_uM
Phe_uM
Trp_uM
Val_uM
Orn_uM
Cit_uM
Tau_uM
AABA_uM
BABA_uM
GABA_uM
Sum_protAAs_uM
Sum_nonprotAAs_uM
Sum_AAs_uM
TASTE1
TASTE2
TASTE3
TASTE4
EAAs
nEAAs
GLU_mgml
FRU_mgml
SUC_mgml
Sum_sugars_mgml
GLU_uM
FRU_uM
SUC_uM
Sugars_uM
1
0.8
0.6
0.4
0.2
0
-0.2
-0.4
-0.6
-0.8
-1

LIP

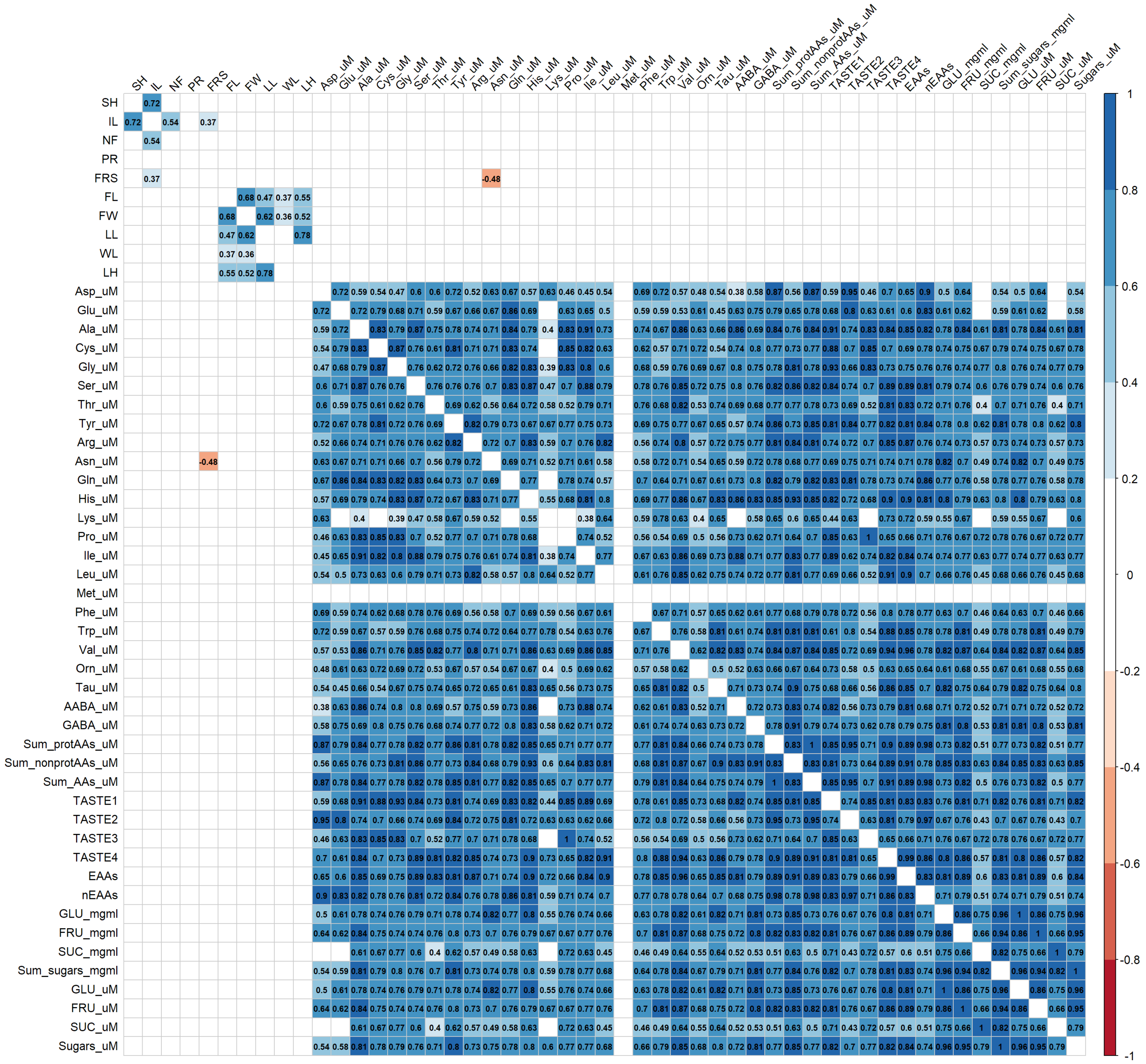
SH
IL
NF
PR
FRS
FL
FW
LL
WL
LH
Asp_uM
Glu_uM
Ala_uM
Cys_uM
Gly_uM
Ser_uM
Thr_uM
Tyr_uM
Arg_uM
Asn_uM
Gln_uM
His_uM
Lys_uM
Pro_uM
Ile_uM
Leu_uM
Met_uM
Phe_uM
Trp_uM
Val_uM
Orn_uM
Tau_uM
AABA_uM
GABA_uM
Sum_protAAs_uM
Sum_nonprotAAs_uM
Sum_AAs_uM
TASTE1
TASTE2
TASTE3
TASTE4
EAAs
nEAAs
GLU_mgml
FRU_mgml
SUC_mgml
Sum_sugars_mgml
GLU_uM
FRU_uM
SUC_uM
Sugars_uM
1
0.8
0.6
0.4
0.2
0
-0.2
-0.4
-0.6
-0.8
-1

SOS

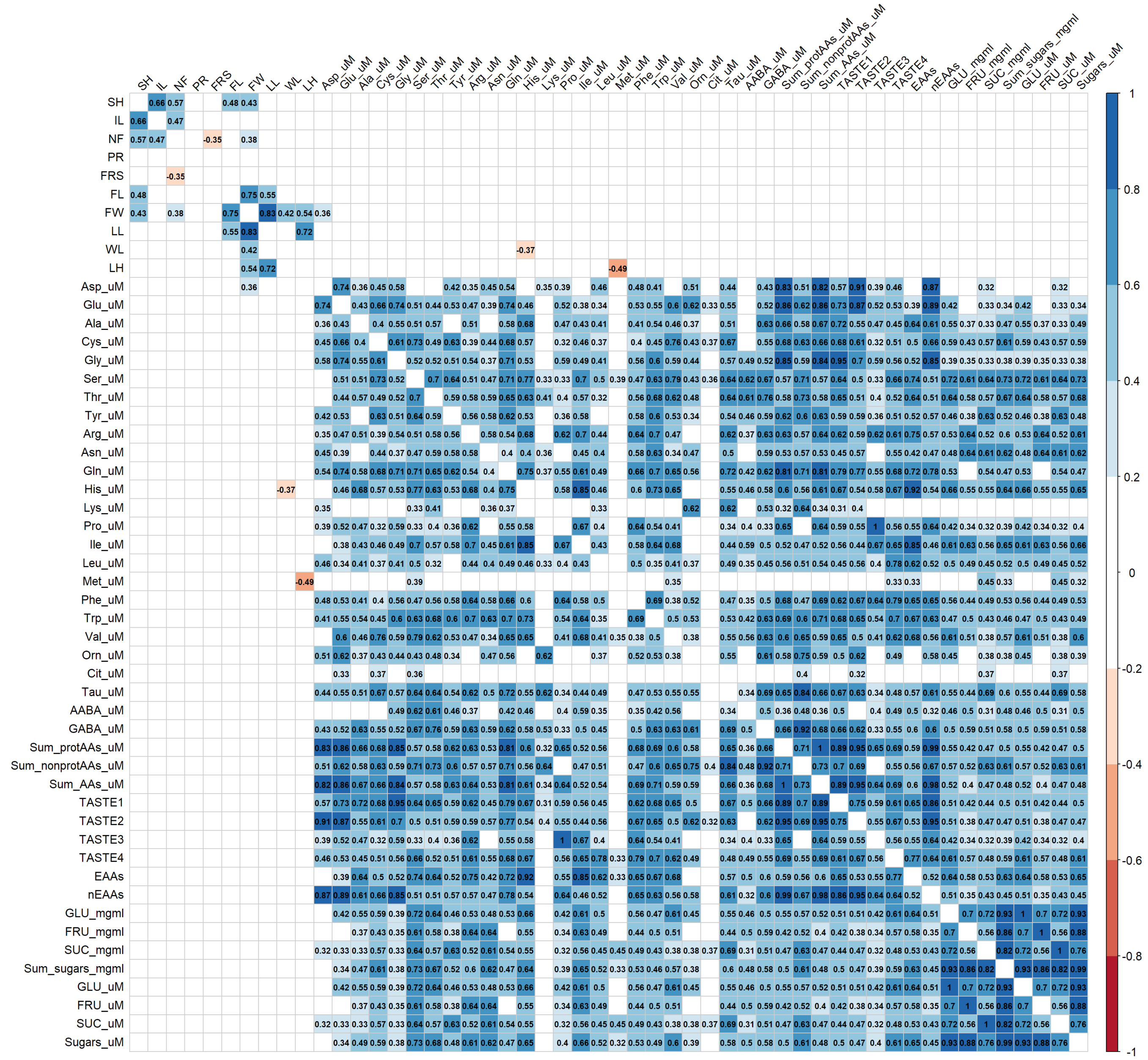
SH
IL
NF
PR
FRS
FL
FW
LL
WL
LH
Asp_uM
Glu_uM
Ala_uM
Cys_uM
Gly_uM
Ser_uM
Thr_uM
Tyr_uM
Arg_uM
Asn_uM
Gln_uM
His_uM
Lys_uM
Pro_uM
Ile_uM
Leu_uM
Met_uM
Phe_uM
Trp_uM
Val_uM
Orn_uM
Cit_uM
Tau_uM
AABA_uM
GABA_uM
Sum_protAAs_uM
Sum_nonprotAAs_uM
Sum_AAs_uM
TASTE1
TASTE2
TASTE3
TASTE4
EAAs
nEAAs
GLU_mgml
FRU_mgml
SUC_mgml
Sum_sugars_mgml
GLU_uM
FRU_uM
SUC_uM
Sugars_uM
1
0.8
0.6
0.4
0.2
0
-0.2
-0.4
-0.6
-0.8
-1

WIG

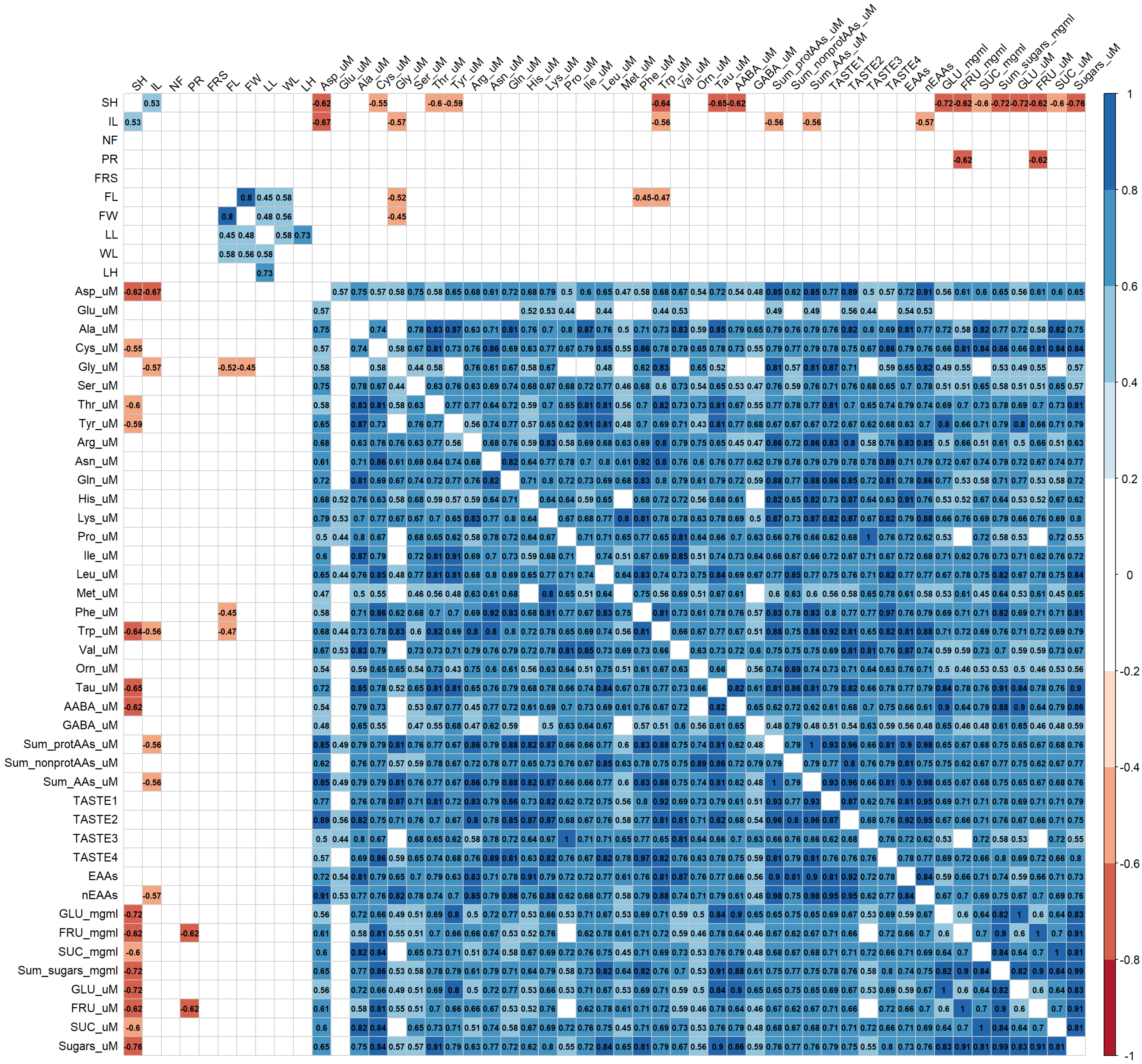
SH
IL
NF
PR
FRS
FL
FW
LL
WL
LH
Asp_uM
Glu_uM
Ala_uM
Cys_uM
Gly_uM
Ser_uM
Thr_uM
Tyr_uM
Arg_uM
Asn_uM
Gln_uM
His_uM
Lys_uM
Pro_uM
Ile_uM
Leu_uM
Met_uM
Phe_uM
Trp_uM
Val_uM
Orn_uM
Tau_uM
AABA_uM
GABA_uM
Sum_protAAs_uM
Sum_nonprotAAs_uM
Sum_AAs_uM
TASTE1
TASTE2
TASTE3
TASTE4
EAAs
nEAAs
GLU_mgml
FRU_mgml
SUC_mgml
Sum_sugars_mgml
GLU_uM
FRU_uM
SUC_uM
Sugars_uM
1
0.8
0.6
0.4
0.2
0
-0.2
-0.4
-0.6
-0.8
-1
